# Supplementary material for: Engineering oxygen nonbonding states in high entropy hydroxides for scalable water oxidation
Source: Nat Commun. 2025 Jul 18;16:6624. doi: 10.1038/s41467-025-61766-2 (PMC12274509; doi:10.1038/s41467-025-61766-2)
Supplement: Supplementary file 1 — Supplementary Information [file 41467_2025_61766_MOESM1_ESM.pdf]

Supplementary Information for

Engineering Oxygen Nonbonding States in High Entropy Hydroxides for Scalable Water Oxidation

Fangqing Wang[1*], Liu Feng[2], Mingwei Zhang[1,2] Hailin Cong[1,3*]

[1] School of Materials Science and Engineering, Shandong University of Technology, Zibo, 255049, PR China

[2] Analytical and Testing Center, Shandong University of Technology, Zibo 255049, PR China

[3] College of Chemistry, Chemical Engineering and Materials Science, Zaozhuang University, Zaozhuang 277160, China

Corresponding author E-mail: conghailin@sdut.edu.cn (Hailin Cong)

wfq970111@163.com (Fangqing Wang)

Supplementary Note 1. Investigate the effect of Na doping on the OER activity of HE LDH.

To investigate the effect of different Na doping amounts on the OER activity of HE LDH, we performed electrochemical performance tests on $Na_{0.025}$-HE LDH, $Na_{0.045}$-HE LDH and $Na_{0.065}$-HE LDH, and the results are shown in **Fig. S1**. $Na_{0.045}$-HE LDH exhibited the most excellent OER activity, with an over potential of 176 mV at 10 mA cm^{-2} (**Fig. S1a**), which is lower than $Na_{0.025}$-HE LDH (232 mV) and $Na_{0.065}$-HE LDH (277 mV). In addition, $Na_{0.045}$-HE LDH exhibits the fastest reaction kinetics (**Fig. S1b**), the large electrocatalytically active surface area (**Fig. S1c**) and the smallest charge transfer resistance (**Fig. S1d**). Too little Na doping may lead to incomplete lattice oxygen activation and thus limited enhancement of OER activity. Too much Na doping may lead to a relative decrease in active sites. Therefore, $Na_{0.045}$-HE LDH (labelled as Na-HE LDH in the manuscript) was chosen as the target catalyst to compare with pristine HE LDH to investigate the intrinsic mechanism of Na ions on the activation of HE LDH.

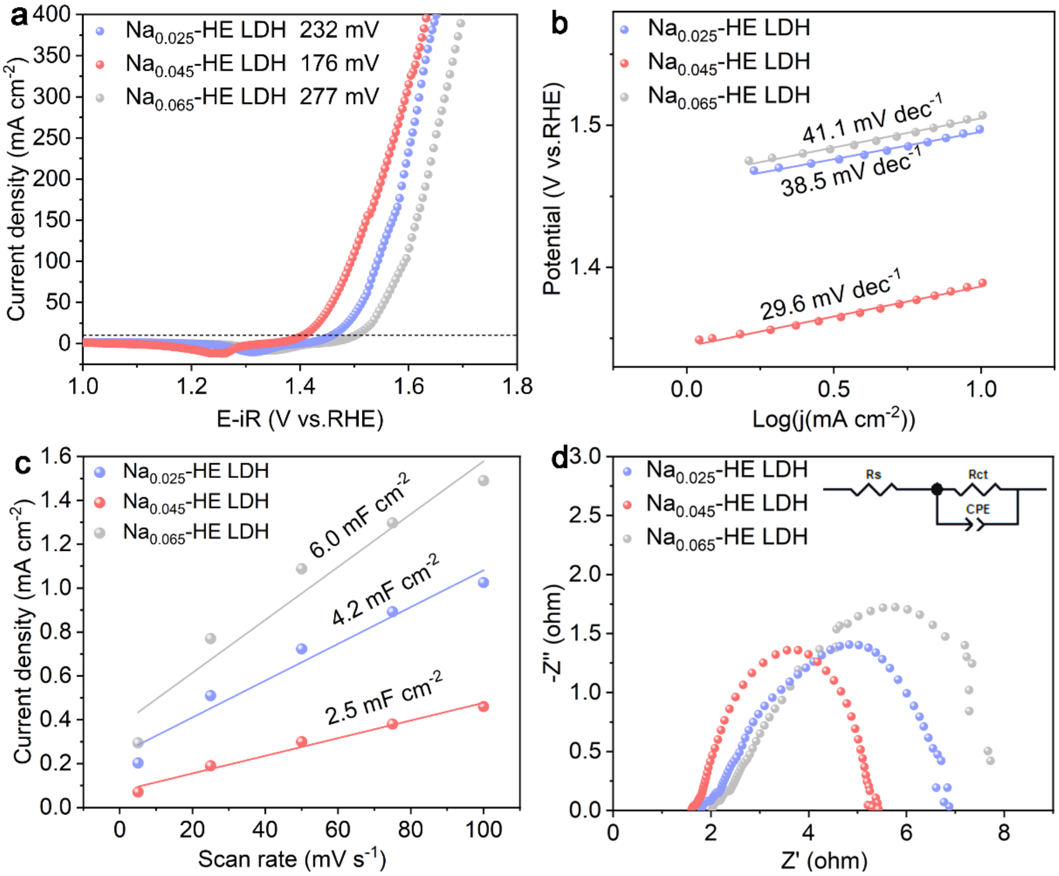

Fig. S1. OER performance of $Na_{0.025}$-HE LDH, $Na_{0.045}$-HE LDH and $Na_{0.065}$-HE LDH in 1.0 M KOH. (a) LSV curves with 95% iR correction, the solution resistance is 1.8 Ω, (b)Tafel plots, (c) C_{dl} plots and (d) EIS curves.

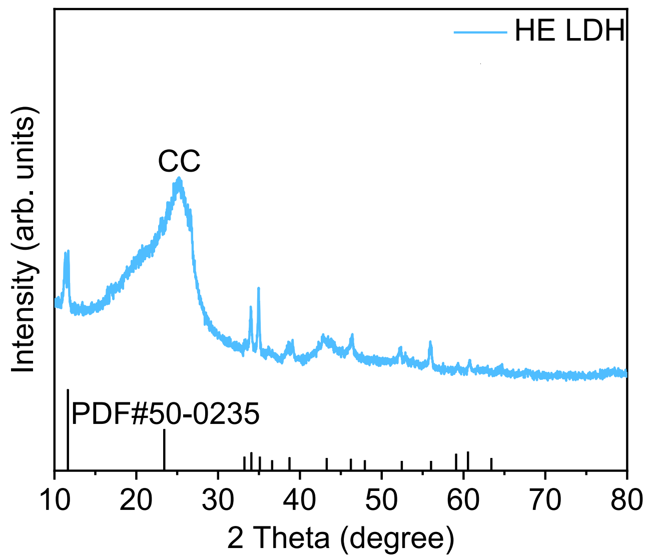

Fig. S2. XRD pattern of HE LDH.

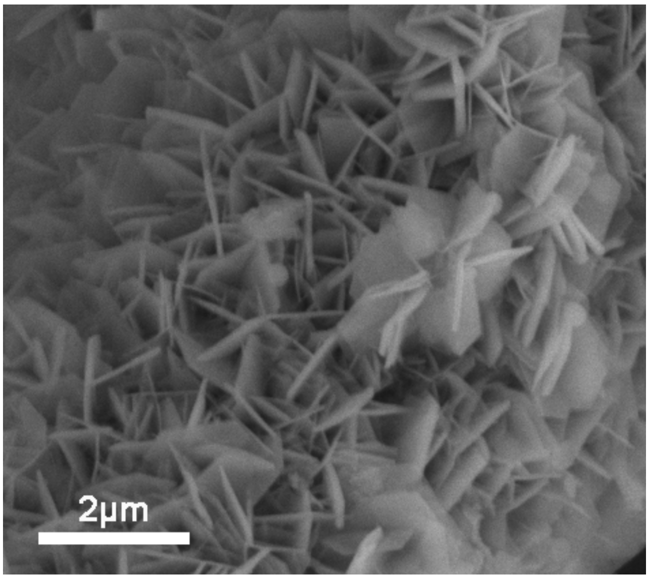

Fig. S3. SEM images of HE LDH.

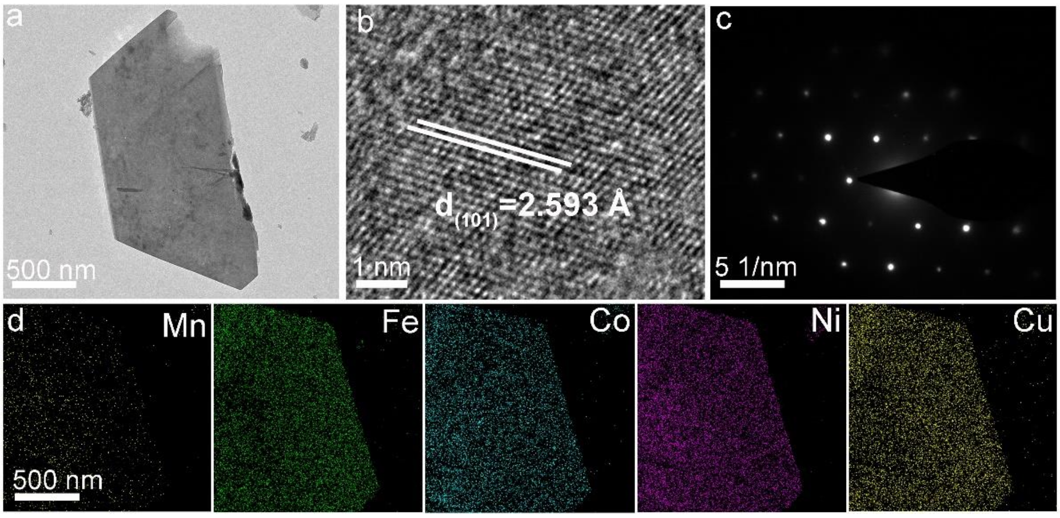

Fig. S4. (a) TEM image. (b) HRTEM image. (c) SAED pattern. (d) EDS elemental mapping of HE LDH.

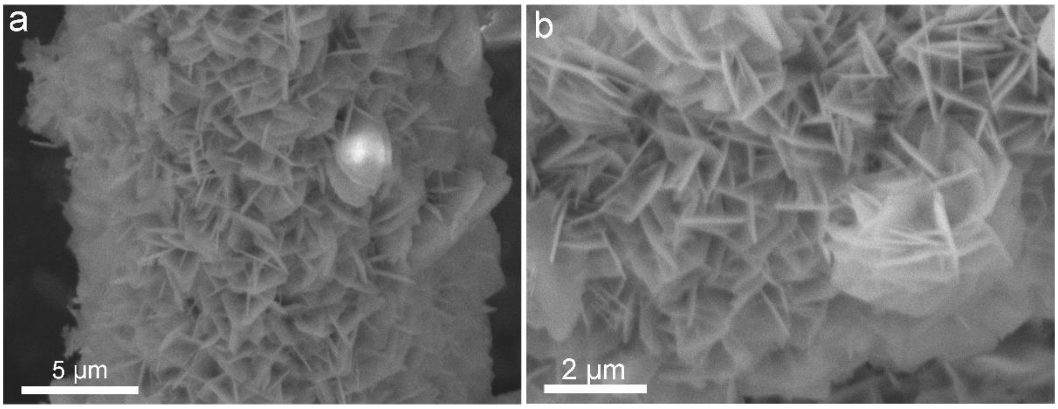

Fig. S5. SEM images of Na-HE LDH at different magnifications.

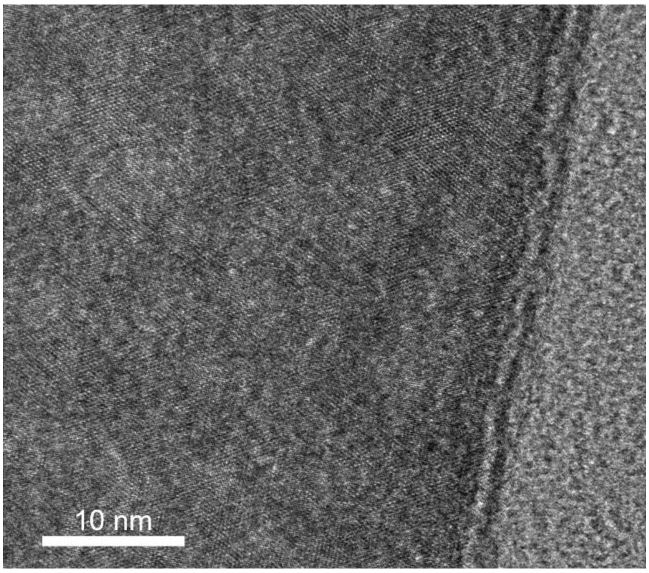

Fig. S6. High-resolution TEM images of Na-HE LDH before OER test.

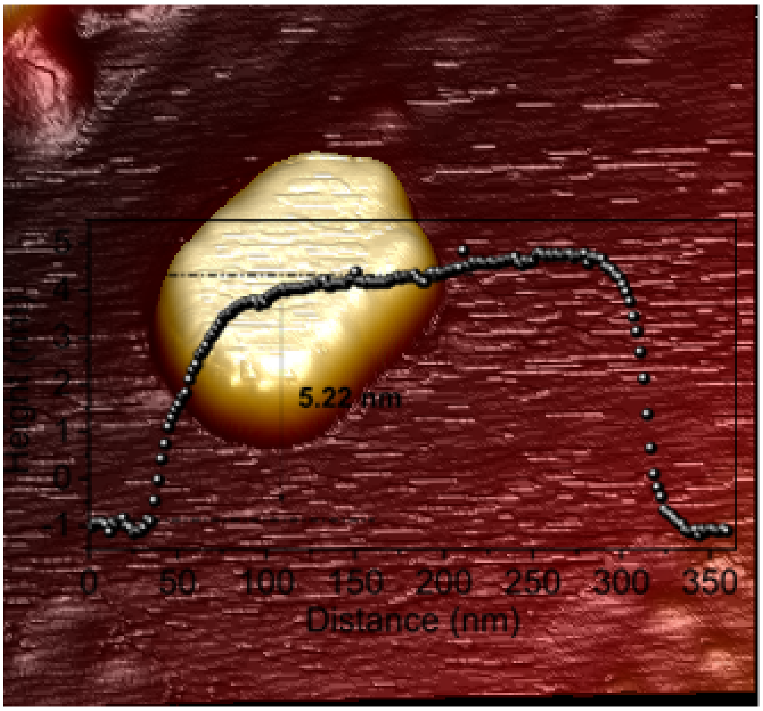

Fig. S7. AFM image of HE LDH.

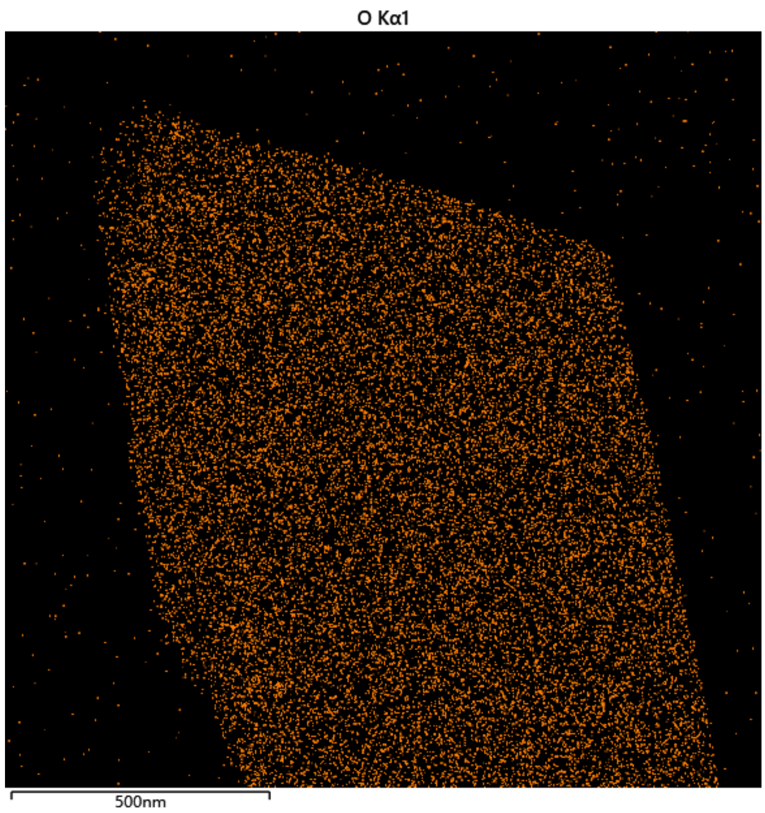

Fig. S8. EDS-Mapping of O of Na-HE LDH.

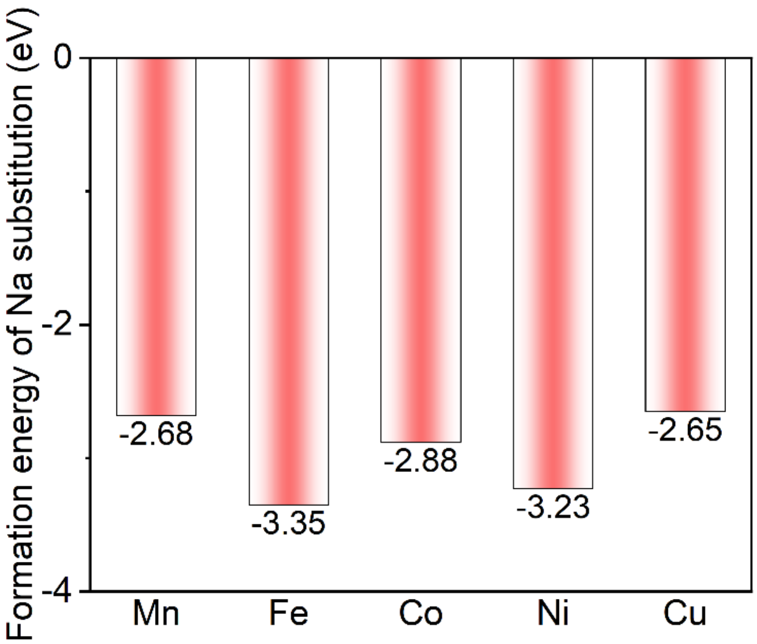

Fig. S9. Calculated the Na substitution formation energies via DFT. Na is easier to replace Fe (-3.35 eV) than Mn (-2.68 eV), Co (-2.88 eV), Ni (-3.23 eV) and Cu (-2.65 eV).

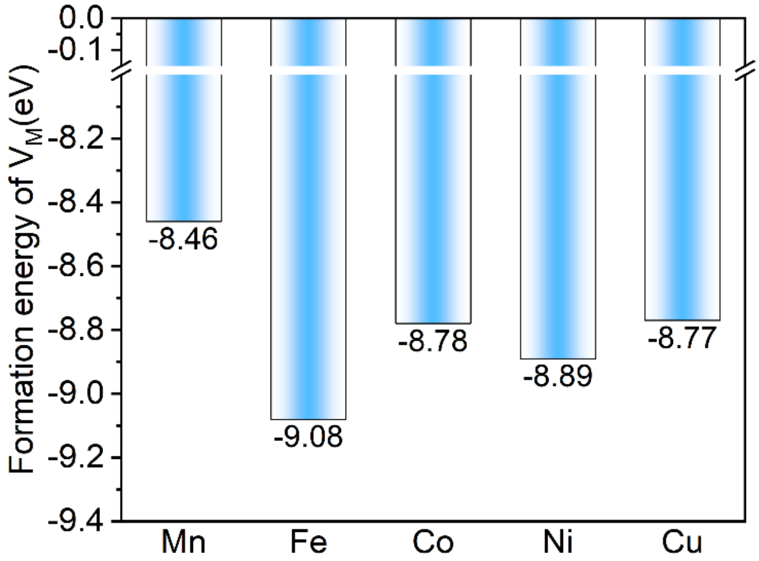

Fig. S10. Metal vacancy formation energy (V_M) of HE LDH. From the thermodynamic point of view, Fe vacancies are more likely to form.

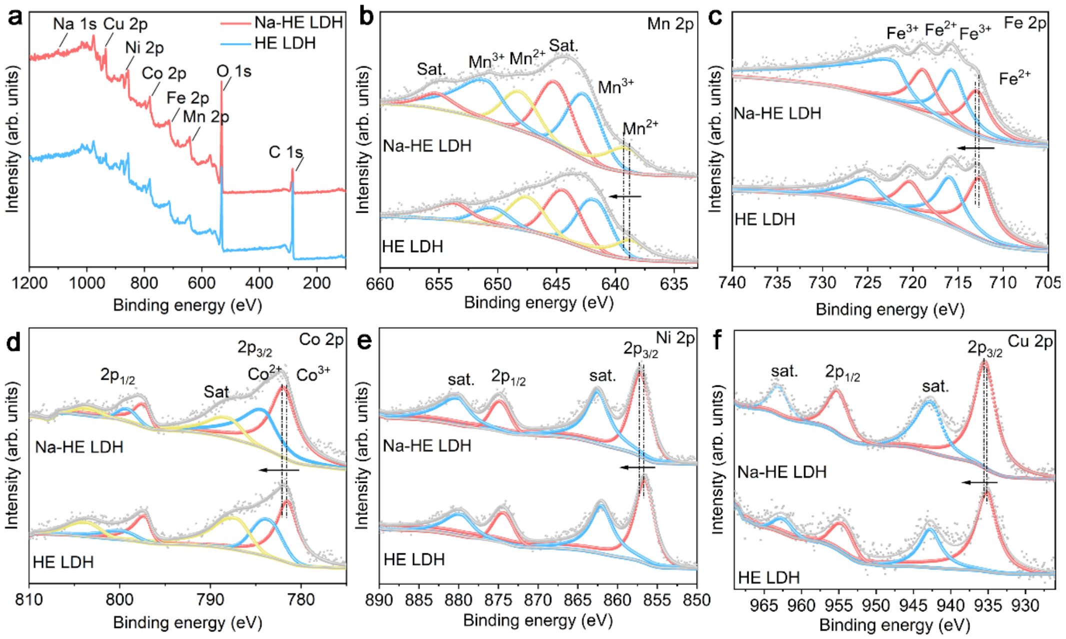

Fig. S11. XPS spectra of (a) Survey, (b) Mn 2p, (c) Fe 2p, (d) Co 2p, (e) Ni 2p, (f) Cu 2p of Na-HE LDH and HE LDH.

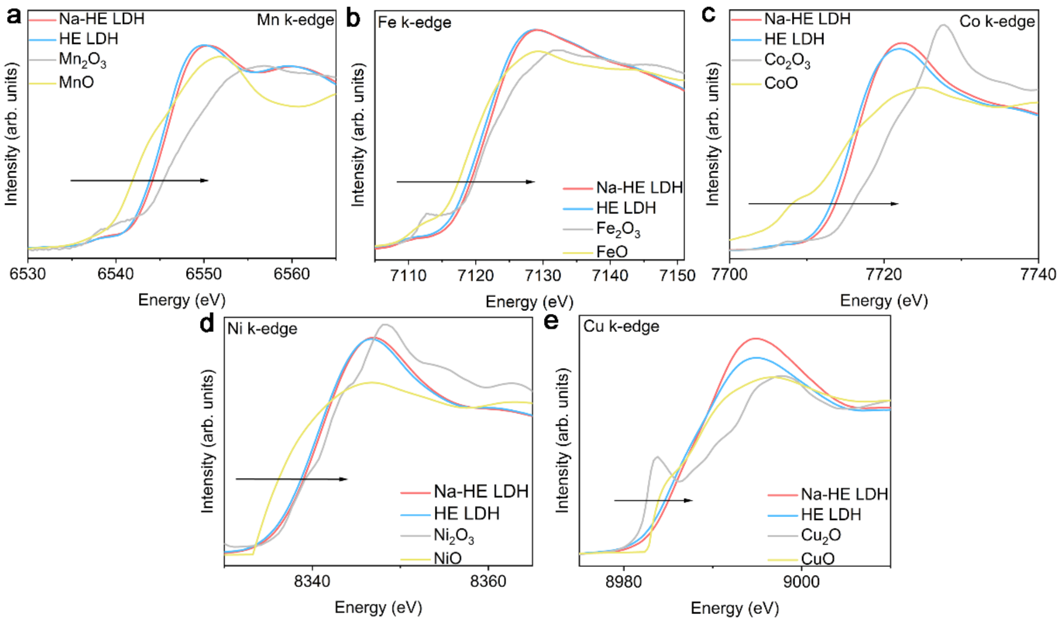

Fig. S12. Normalized *K*-edge XAFS spectra of (a) Mn, (b) Fe, (c) Co, (d) Ni and (e) Cu elements. Compared with HE LDH, all the absorption edges of Mn, Fe, Co, Ni and Cu in Na-HE LDH shift to higher energies, implying an increased valence of 3d transition metals.

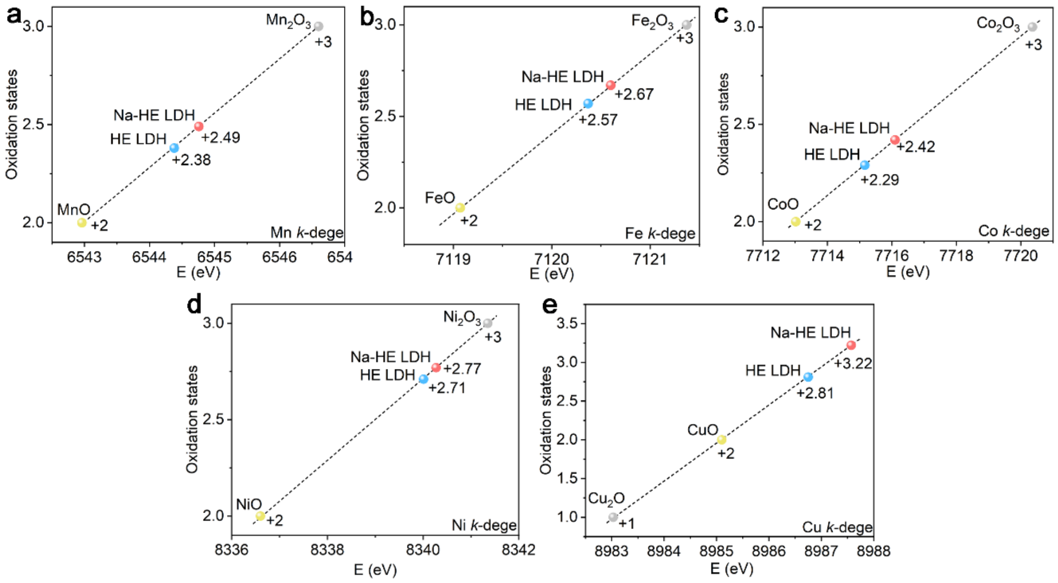

Fig. S13. Correlation between the metal oxidation states and the energy position of the XANES spectra.

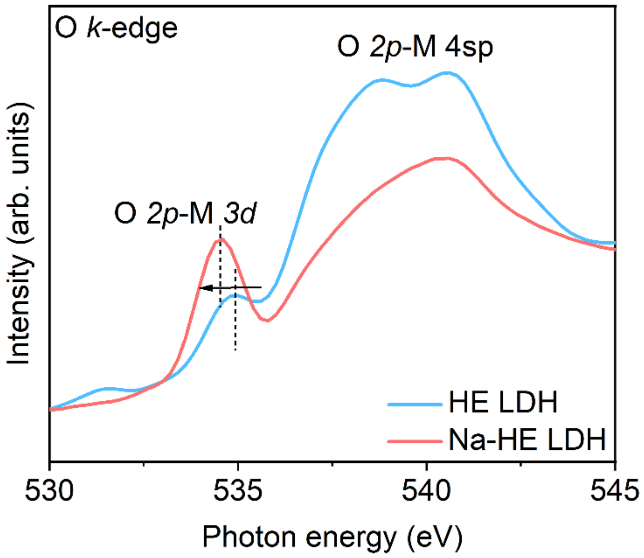

Fig. S14. O K-edge soft X-ray absorption spectroscopies (sXAS) of Na-HE LDH and HE LDH.

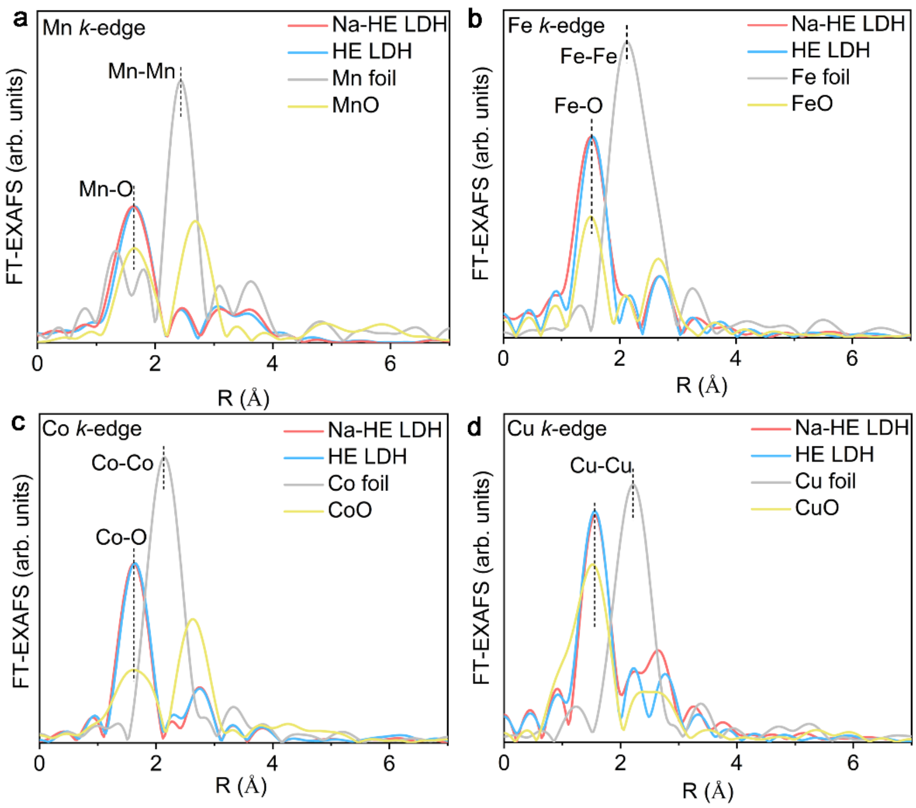

Fig. S15. FT-EXAFS spectra and fitting results of Mn, Co, Ni and Cu elements. The changes in Mn-O, Co-O, Ni-O and Cu-O of Na-HE LDH and HE LDH are almost negligible.

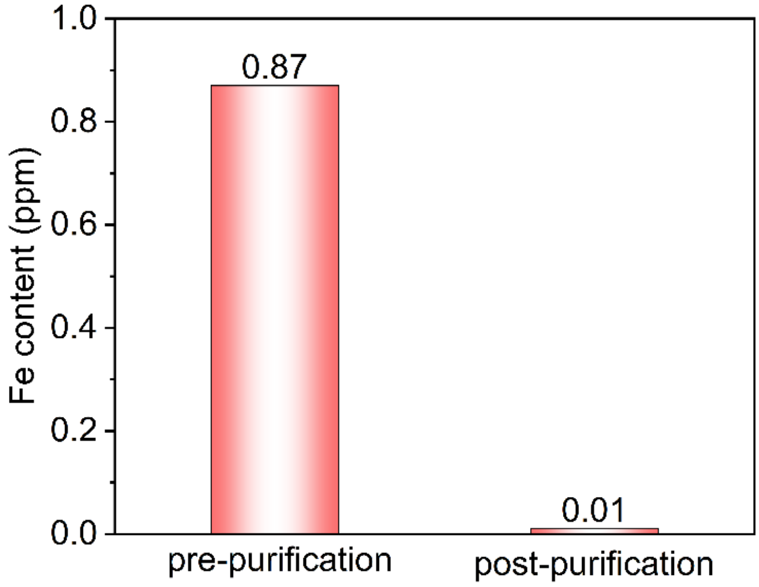

Fig. S16. Fe content in the electrolyte before and after purification, determined by ICP-MS.

Supplementary Note 2. Investigate the effect of Na doping on the OER activity of conventional binary LDH.

To elucidate the reason for the improved performance, we prepared conventional binary LDHs (NiFe LDH, NiCo LDH, NiCu LDH, and NiMn LDH) and Na-doped binary LDHs (Na-NiFe LDH, Na-NiCo LDH, Na-NiCu LDH, and Na-NiMn LDH) and compared them with the HE LDH and Na-HE LDH (**Fig. S16**). The LSV curves (**Fig. S17**) show that Na doping (Na-NiFe LDH (223 mV), Na-NiCo LDH (284 mV), Na-NiCu LDH (317 mV) and Na-NiMn LDH (326 mV)) significantly enhances the OER activities of the conventional binary LDHs (NiFe LDH (234 mV), NiCo LDH (307 mV), NiCu LDH (323 mV) and NiMn LDH (337 mV). Whereas, the activity of HE LDH (330 mV, **Fig. 4a**) is lower than that of conventional binary LDH. this suggests that the improved performance is due to the introduction of Na ions and not due to the high entropy structure.

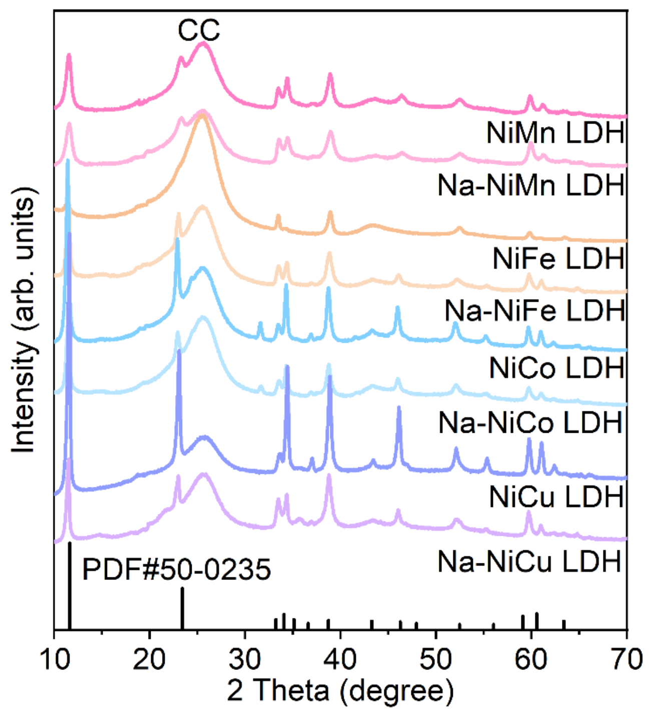

Fig. S17 XRD patterns of NiMn LDH, NiFe LDH, NiCo LDH, NiCu LDH, Na-NiMn LDH, Na-NiFe LDH, Na-NiCo LDH and Na-NiCu LDH.

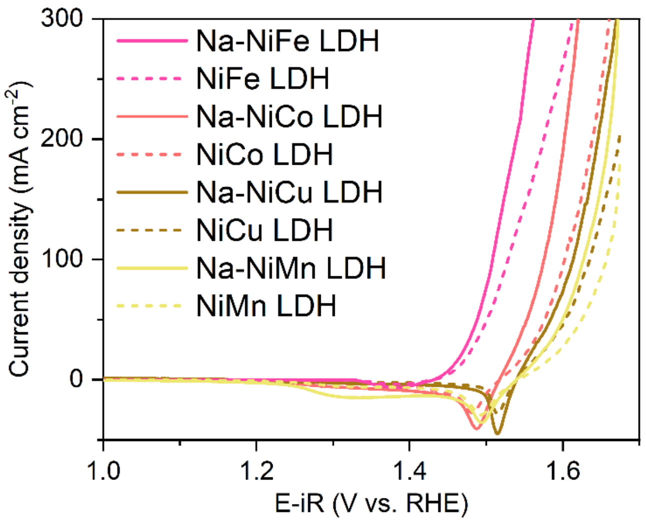

Fig. S18 LSV curves of NiMn LDH, NiFe LDH, NiCo LDH, NiCu LDH, Na-NiMn LDH, Na-NiFe LDH, Na-NiCo LDH and Na-NiCu LDH with 95% iR correction, the solution resistance is 1.8 Ω.

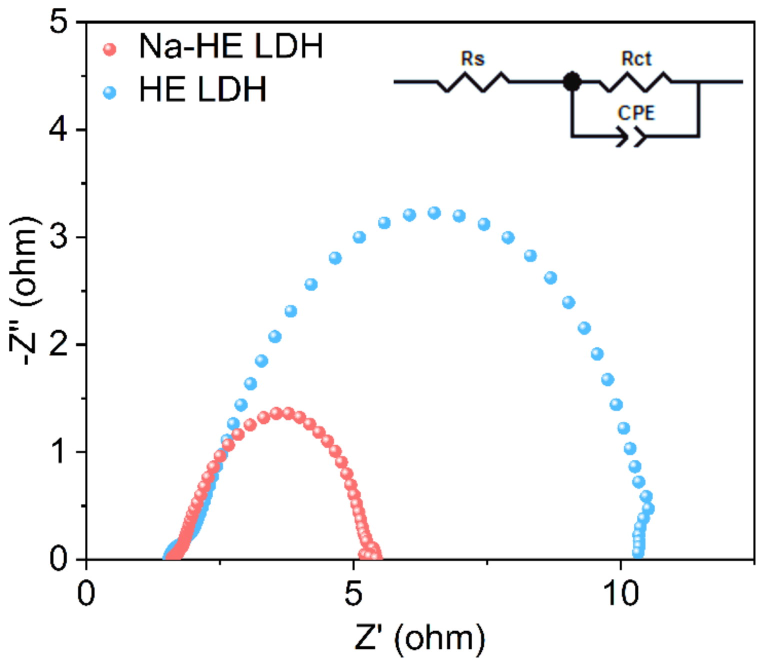

Fig. S19. EIS curves of Na-HE LDH and HE LDH (inset is equivalent circuit).

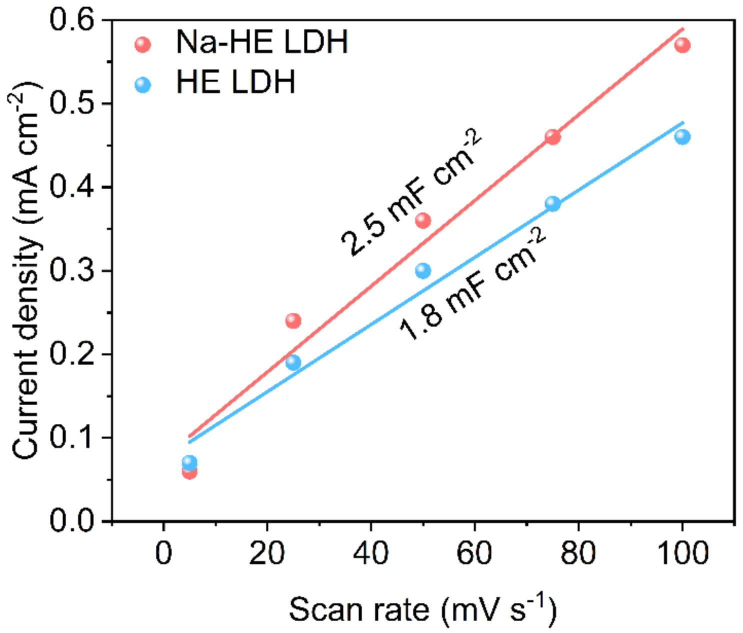

Fig. S20. C_{dl} curves of the Na-HE LDH and HE LDH.

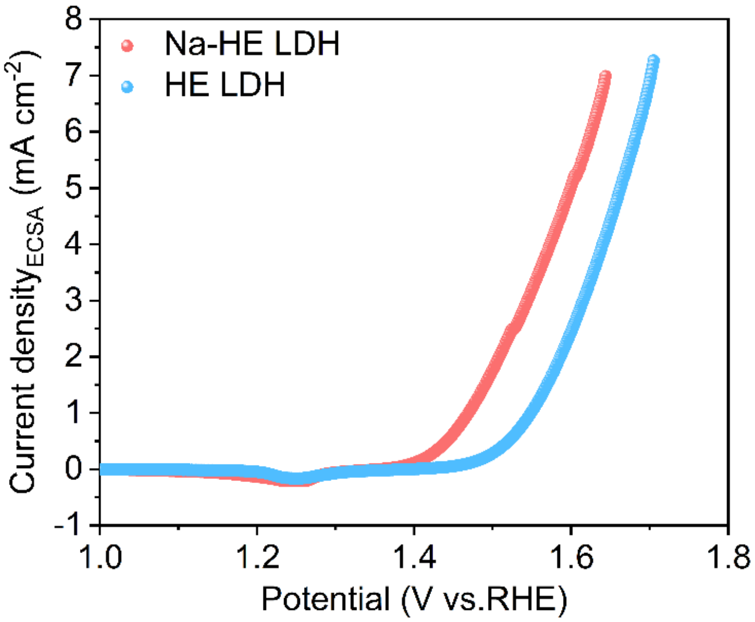

Fig. S21. LSV curves normalized by ECSA of the Na-HE LDH and HE LDH.

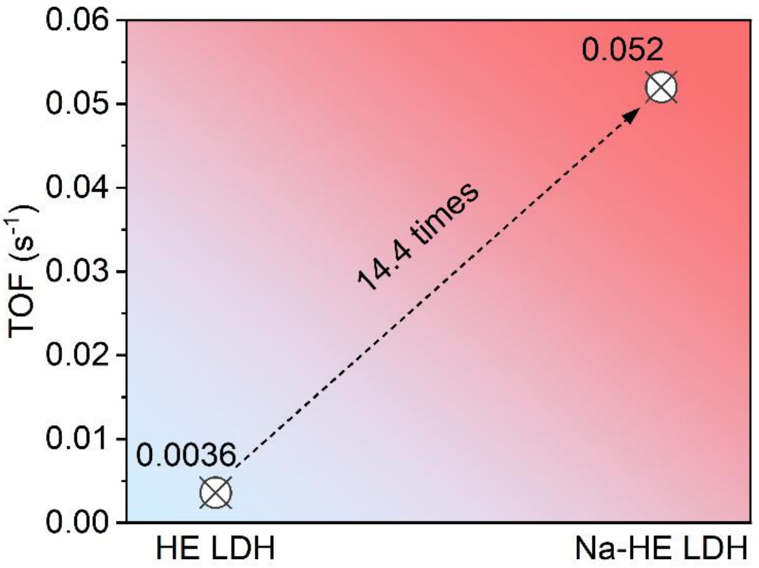

Fig. S22. Turnover frequency (TOF) at 250 mV for Na-HE LDH and HE LDH. O is the active site in Na-HE LDH and Ni is the active site in HE LDH.

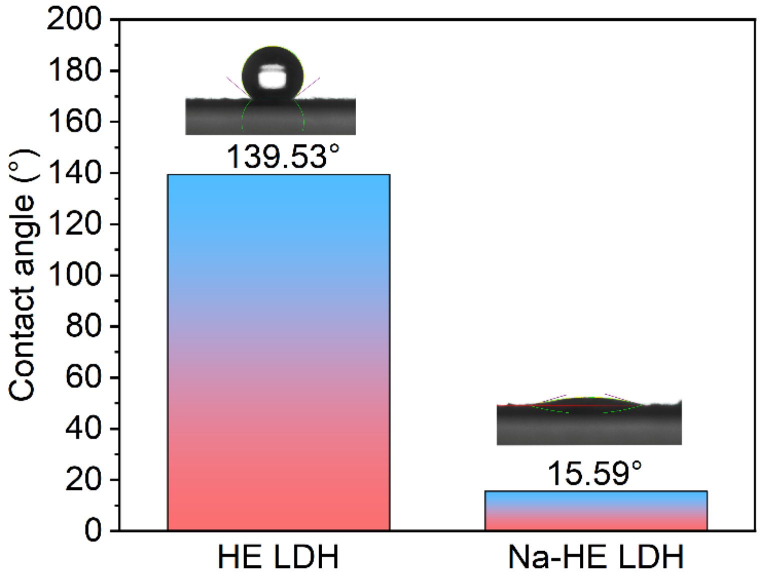

Fig. S23. Contact angle of liquid drop on catalyst surface.

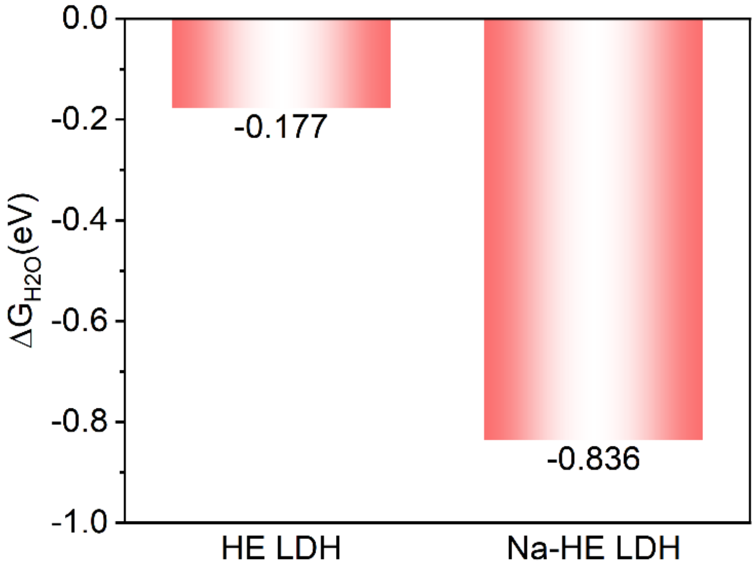

Fig. S24. Calculated adsorption free energy diagrams on the HE LDH (Ni as active site) and Na-HE LDH (O as active site).

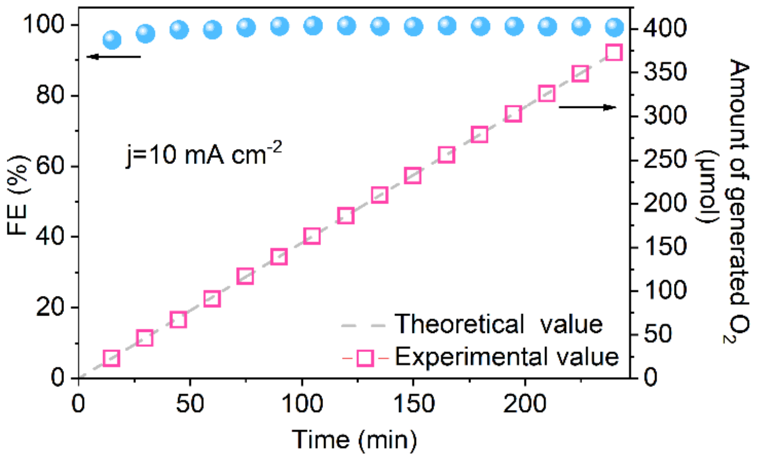

Fig. S25. The FE of Na-HE LDH as a function of reaction time determined by the water displacement method at potential of 1.41 V vs. RHE.

FE determined by water displacement method. The FE was calculated by the equation as follows:

$$FE\ (\%) = \text{Experimental of } O_2 / \text{Theoretical of } O_2 \times 100\%$$

The theoretical amount of O_2 gas (n) was calculated by Faraday's law:

$$N=(i\times t)/(z\times F)$$

where n, is moles, t is the OER reaction time (s), i is the current (A), z is electron transfer number of OER (z=4) and F is the Faraday constant. The experimental amount of O_2 (n) gas was determined by a water-displacement method.

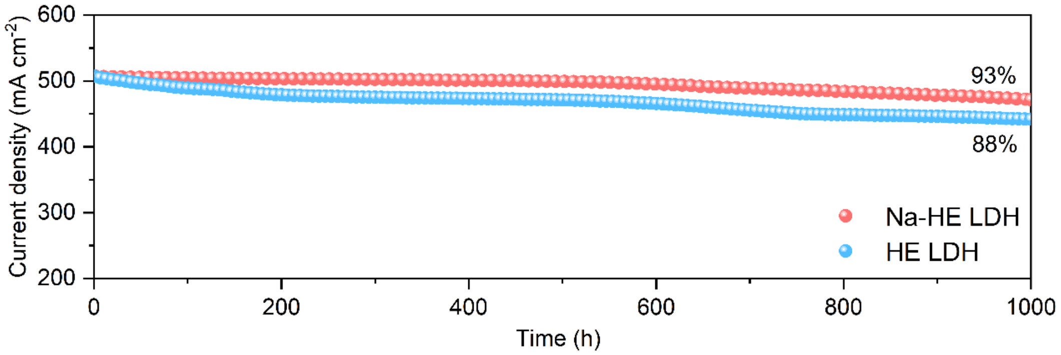

Fig. S26. i-t measurement for Na-HE LDH and HE LDH at 1.65 V vs. RHE and 1.75 V vs. RHE.

Supplementary Note 3. Exploring the importance of high entropy structures for OER stability.

High-entropy structures have high stability due to high-entropy effects and hysteretic diffusion effects, which are essential for catalysts to sustainably undergo OER. To elucidate the necessity of high-entropy structures, we performed stability tests on conventional binary LDHs and compared them with HE LDHs. The results are shown in **Fig. S26**, where NiFe LDH was continuously stable at ~500 mA cm^{-2} for 55 h, NiCo LDH, NiCu LDH, NiMn LDH were rapidly deactivated at the early stage of the stability test, whereas HE LDH was continuously operated at ~500 mA cm^{-2} for 1000 h. This confirmed the necessity of high-entropy structure for long-term OER, especially based on the LOM pathway of OER.

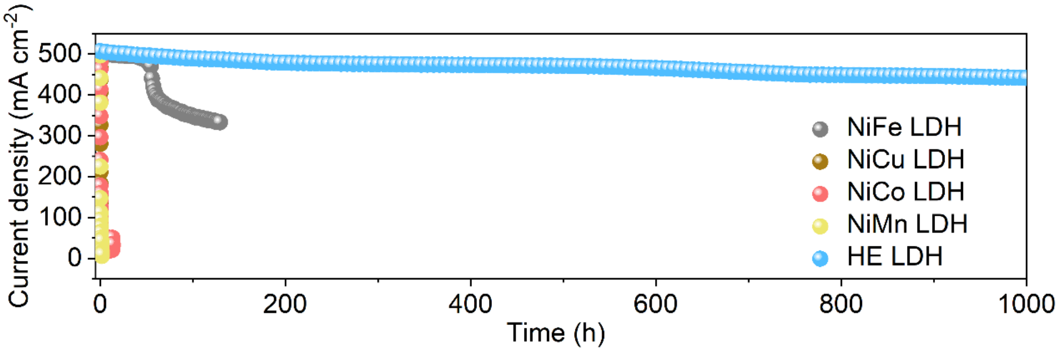

Fig. S27. i-t measurement for NiFe LDH (1.66 V vs. RHE), NiCu LDH (1.76 V vs. RHE), NiCo LDH (1.68 V vs. RHE), NiMn LDH (1.78 V vs. RHE) and HE LDH (1.75 V vs. RHE).

Supplementary Note 4. Investigate the OER activity and stability of Na-HE LDH in 1 M NaOH electrolyte.

To confirm the robust activity and stability of Na-HE LDH in an alkaline environment, we again tested it with 1 M NaOH as the electrolyte. As shown in **Fig. S27**, the overpotential of Na-HE LDH in 1 M NaOH is 204 mV, the Tafel slope is 38.5 mV dec^{-1}, the C_{dl} value is 3.9 mF cm^{-2} and the charge transfer resistance is 3.4 Ω, which is similar to the performance of Na-HE LDH in 1 M KOH. We also tested the long-term stability of Na-HE LDH in 1 M NaOH, and the results showed that Na-HE LDH still has an extremely long durability with almost no decay after 1000 h of continuous operation. In conclusion, Na-HE LDH has excellent OER activity and stability in different alkaline electrolytes and has great potential for use.

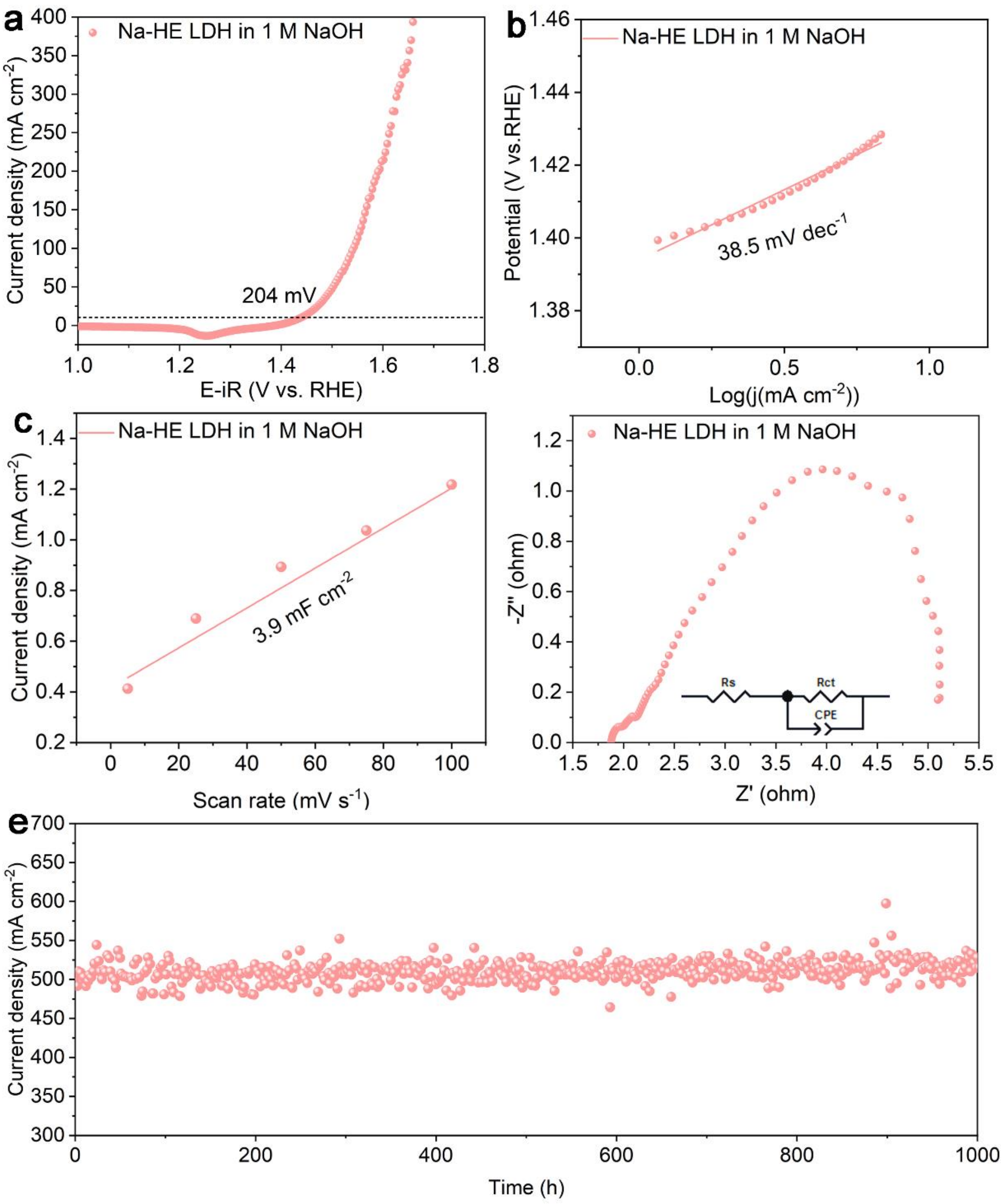

Fig. S28. OER performance of Na-HE LDH OER performance in 1 M NaOH. (a) LSV curve with 95% iR correction, the solution resistance is 1.8 Ω, (b)Tafel plot, (c) C_{dl} plot, (d) EIS curve and (e) i-t measurement at 1.69 V vs. RHE.

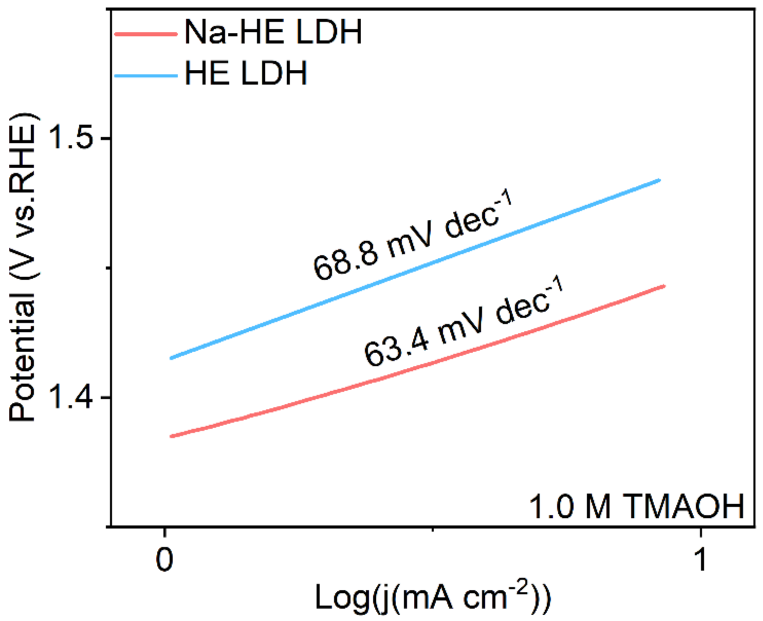

Fig. S29. Tafel plots of Na-HE LDH and HE LDH at 1.0 M TMAOH.

Supplementary Note 5. Investigate the AEMWE properties of Na-HE LDH on nickel foam (NF) substrates.

Considering the high electrical conductivity and alkali resistance of nickel foam, we also reassembled the AEMWE by loading the catalyst on NF for activity and stability tests. As shown in **Fig. S29a-b**, AEMWE based on Pt/C||Na-HE LDH achieves high current densities of 100 mA cm^{-2}, 500 mA cm^{-2} and 1000 mA cm^{-2} at cell voltages as low as 1.51 V, 1.54 V and 1.57 V. This is superior to the use of Pt/C||HE LDH and Pt/C||NiFe LDH electrolysis cells. Here we note that due to the high conductivity of NF, AEMWE with NF as the carrier has higher activity than AEMWE with carbon cloth as the carrier, especially at high current densities. More importantly, the Pt/C|||Na-HE LDH electrolyser with NF as the carrier also showed excellent long-term stability after 1000 h of continuous testing at ~500 mA cm^{-2} (**Fig. S29c**).

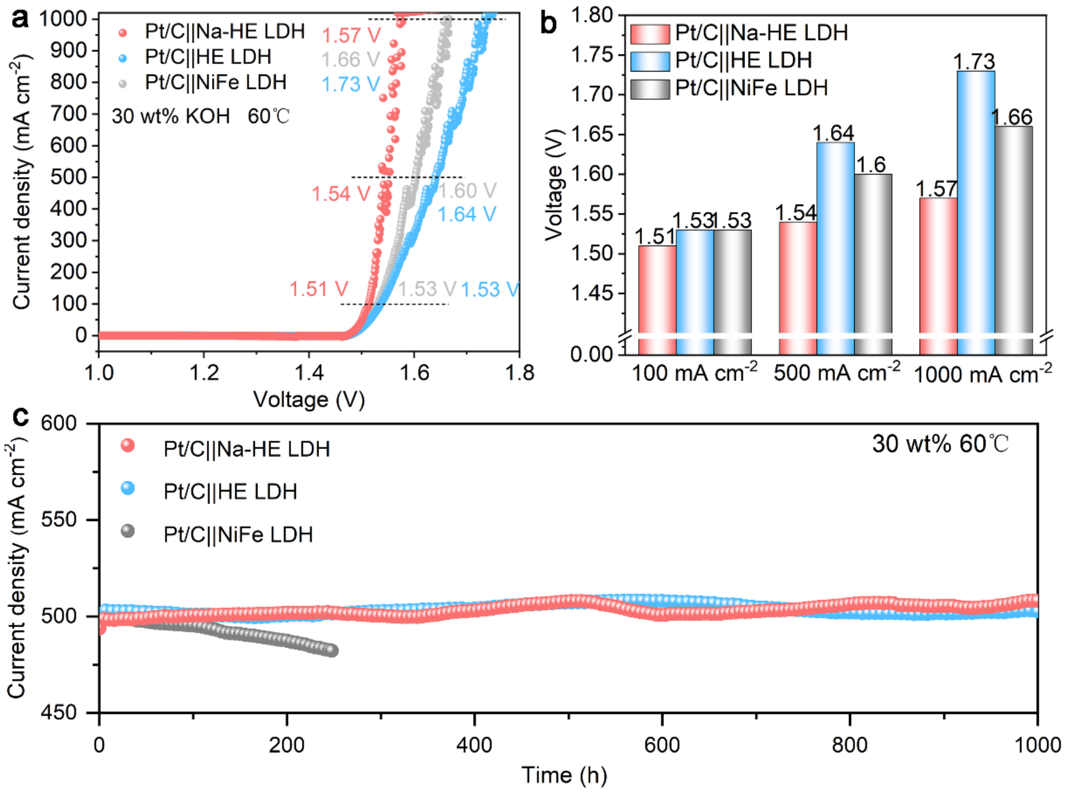

Fig. S30. (a) LSV curves of Pt/C|| Na-HE LDH, Pt/C||HE LDH and Pt/C||NiFe LDH in 30 wt% KOH at 60°C, no iR correction. (b) Comparison of voltage values for Pt/C|| Na-HE LDH, Pt/C||HE LDH and Pt/C||NiFe LDH at different current densities. (c) i-t measurement for Pt/C|| Na-HE LDH, Pt/C||HE LDH and Pt/C||NiFe LDH at 1.55 V vs. RHE, 1.65 V vs. RHE and 1.61 V vs. RHE.

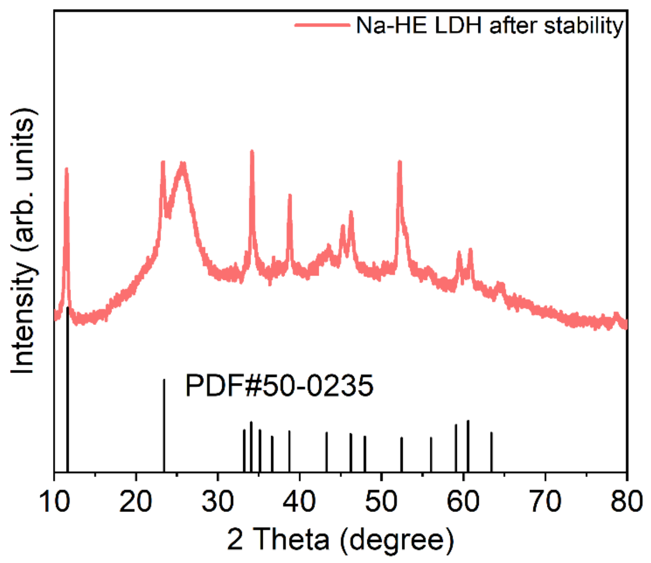

Fig. S31. XRD pattens of Na-HE LDH after long-term OER test.

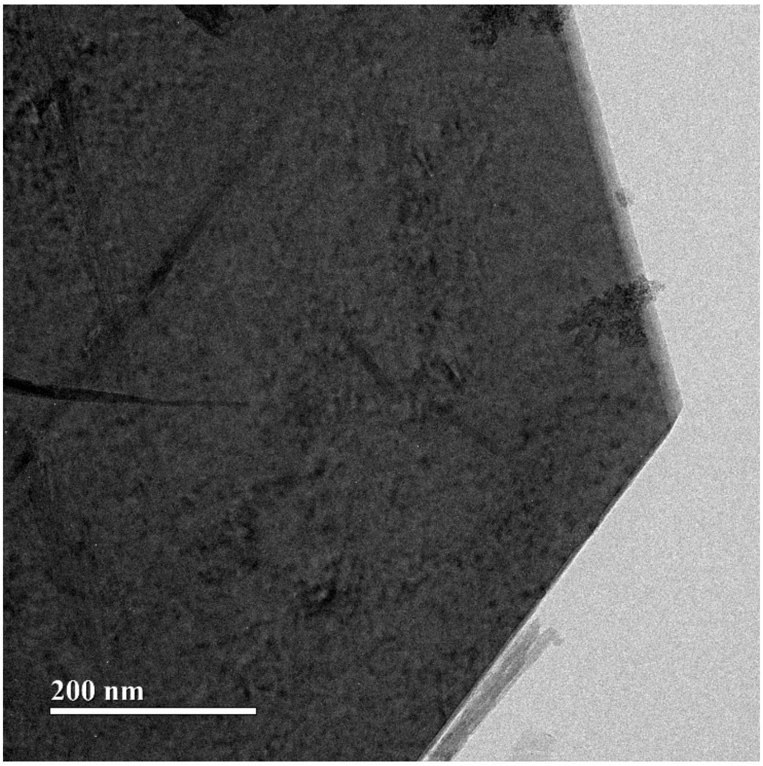

Fig. S32. TEM image of Na-HE LDH after long-term OER test.

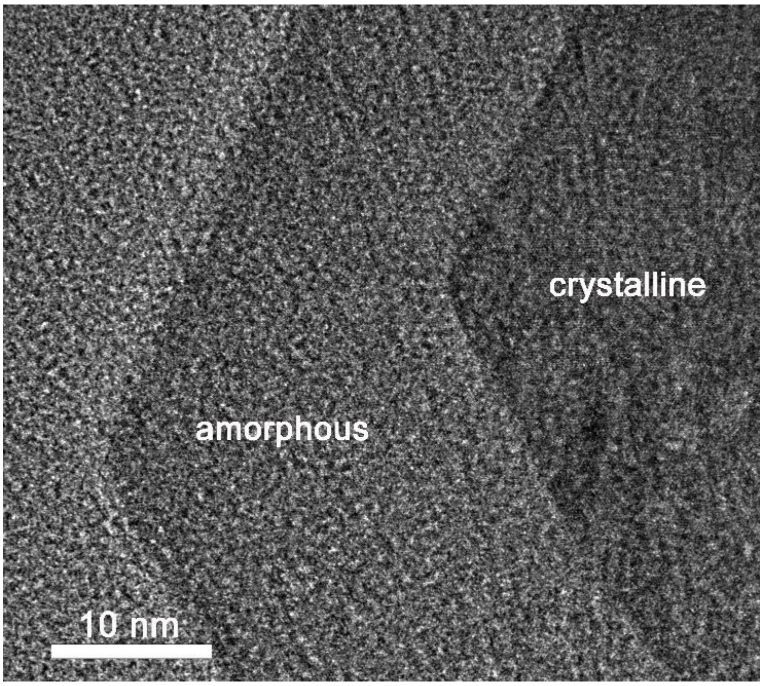

Fig. S33. HRTEM image of Na-HE LDH after long-term OER test.

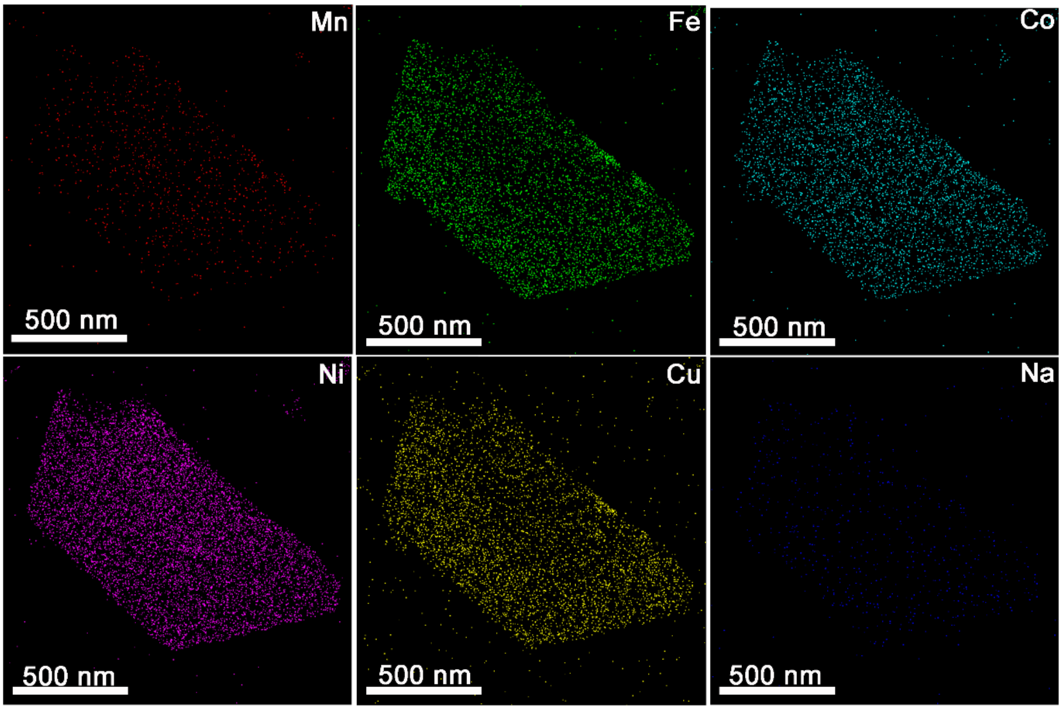

Fig. S34. EDS-Mapping of Na-HE LDH after long-term OER test.

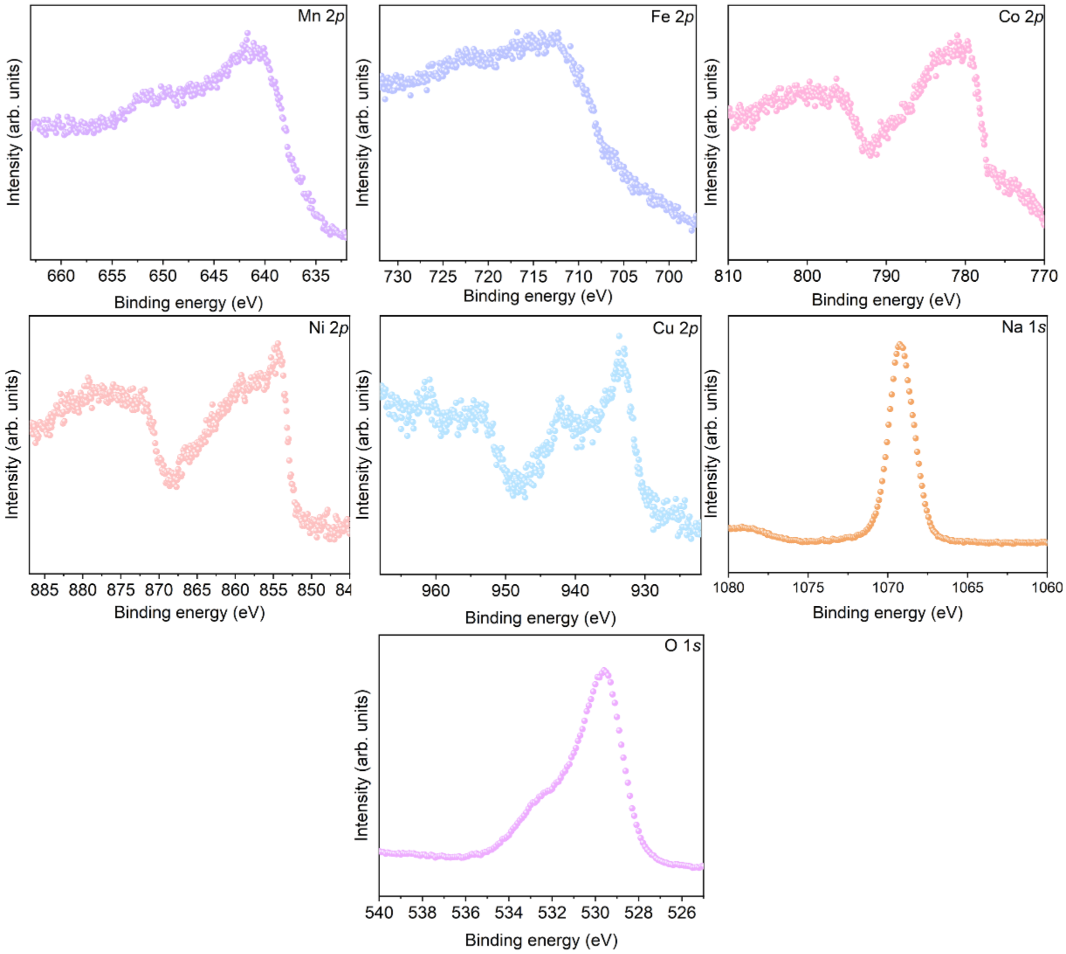

Fig. S35. XPS spectra of Na-HE LDH after long-term OER test.

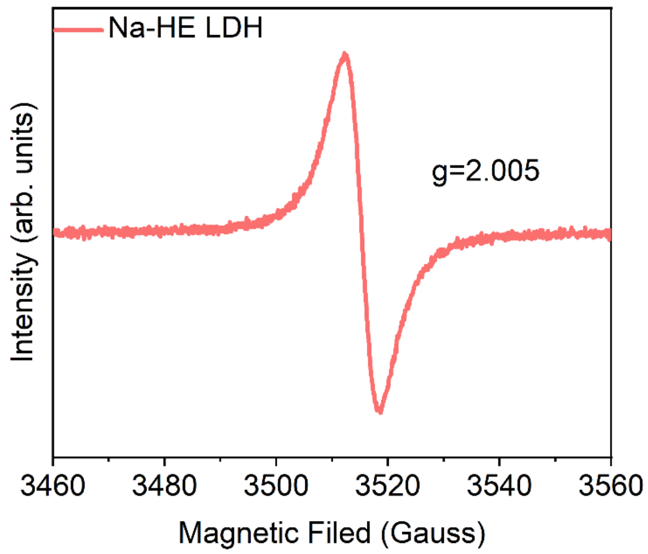

Fig. S36. EPR results of Na-HE LDH after long-term OER test.

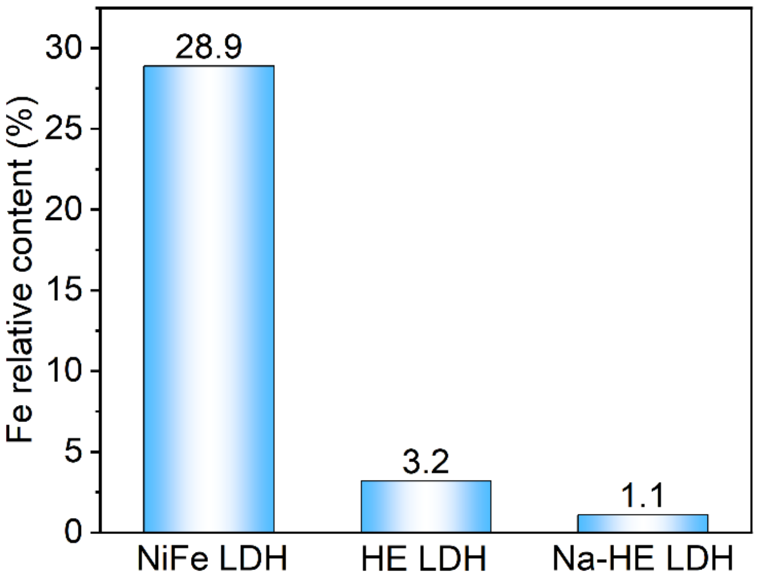

Fig. S37. Fe dissolution contents of NiFe LDH, HE LDH and Na-HE LDH.

After the stability test of NiFe LDH, HE LDH and Na-HE LDH, the proportion of Fe element dissolved in the electrolyte to the Fe element in the original sample, which stands for the Fe dissolution percentage by ICP-MS. The calculation formula as below:

$$\text{Relative content} = \frac{C_d \times V}{C_o \times m}$$

Here C_d (mg L^{-1}) is the dissolved concentration of the Fe element in the electrolyte after stability test, V (L) is the volume of electrolyte during stability test, C_0 (mg kg^{-1}) is the original loading content of the Fe element on the electrode before stability test, and m (kg) is the mass of electrode.

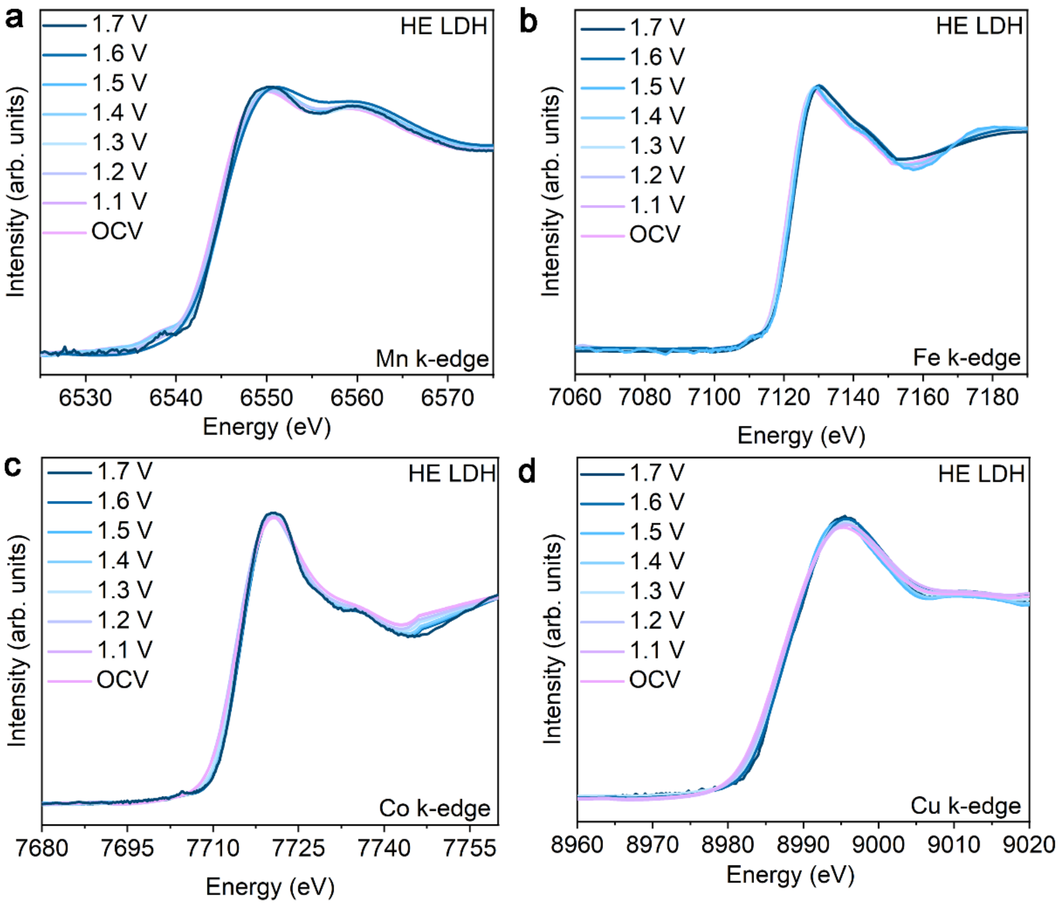

Fig. S38. In-situ Mn, Fe, Co and Cu k-edge XANES spectra of HE LDH under applied potentials of 1.1–1.7 V vs. RHE in 1.0 M KOH.

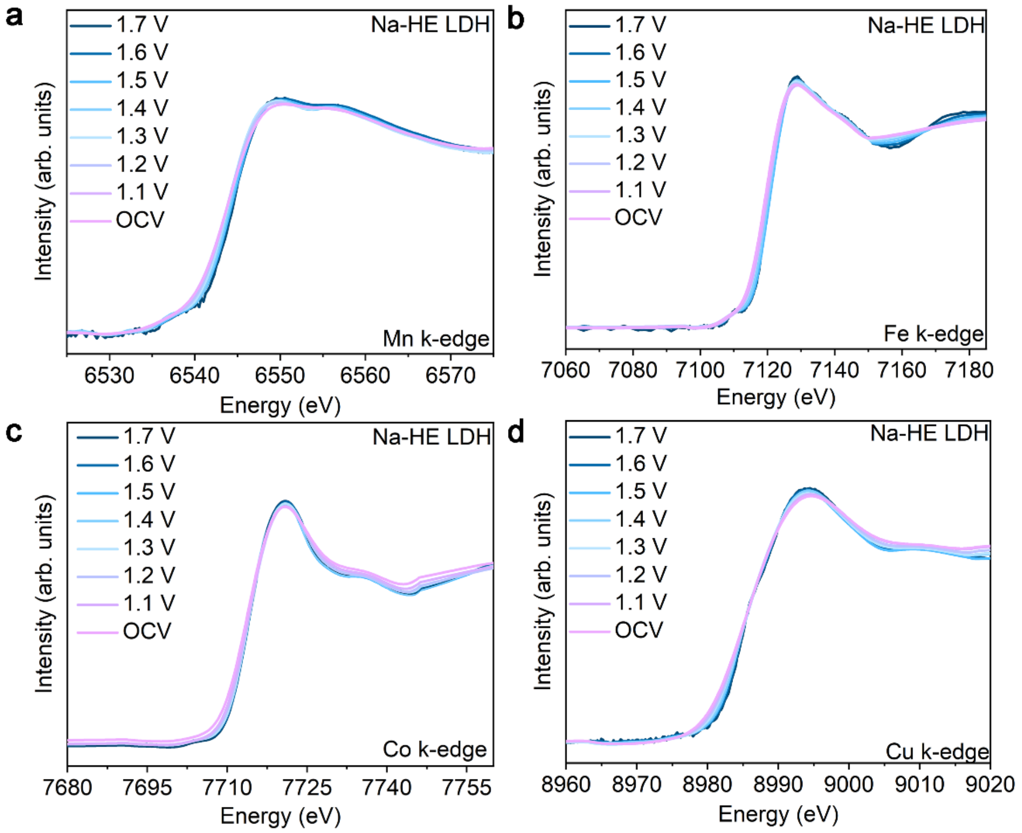

Fig. S39. In-situ Mn, Fe, Co and Cu k-edge XANES spectra of Na-HE LDH under applied potentials of 1.1–1.7 V vs. RHE in 1.0 M KOH.

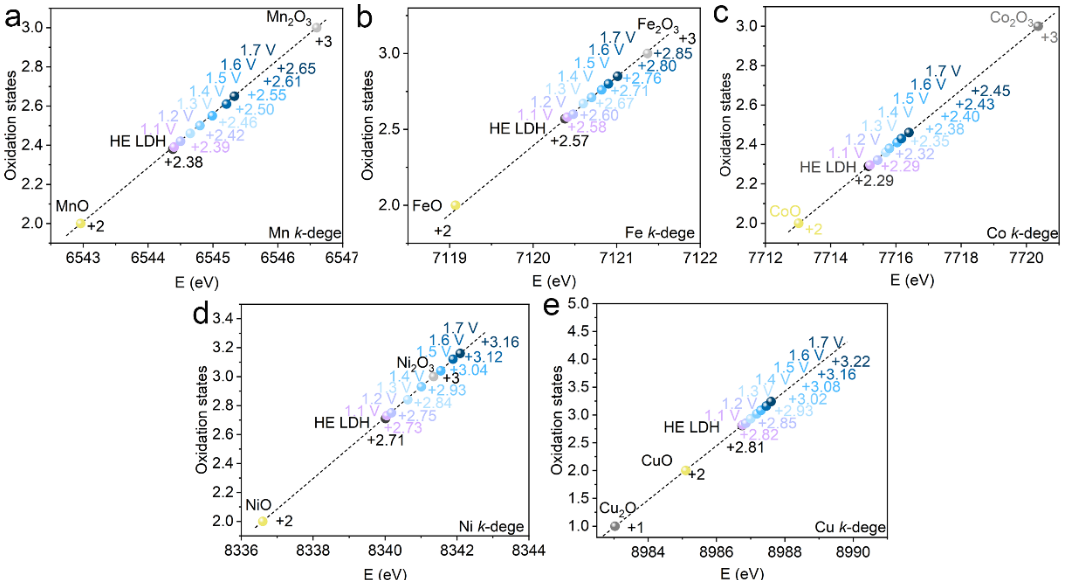

Fig. S40. Variation of metal oxidation state with voltage in HE LDH under applied potentials of 1.1–1.7 V vs. RHE in 1.0 M KOH.

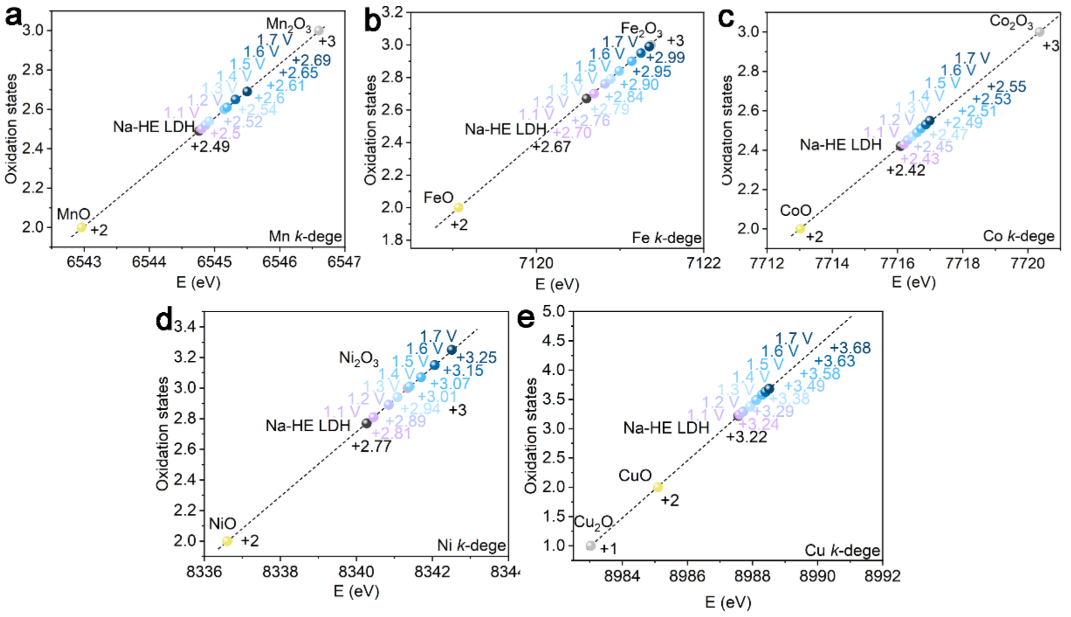

Fig. S41. Variation of metal oxidation state with voltage in Na-HE LDH under applied potentials of 1.1–1.7 V vs. RHE in 1.0 M KOH.

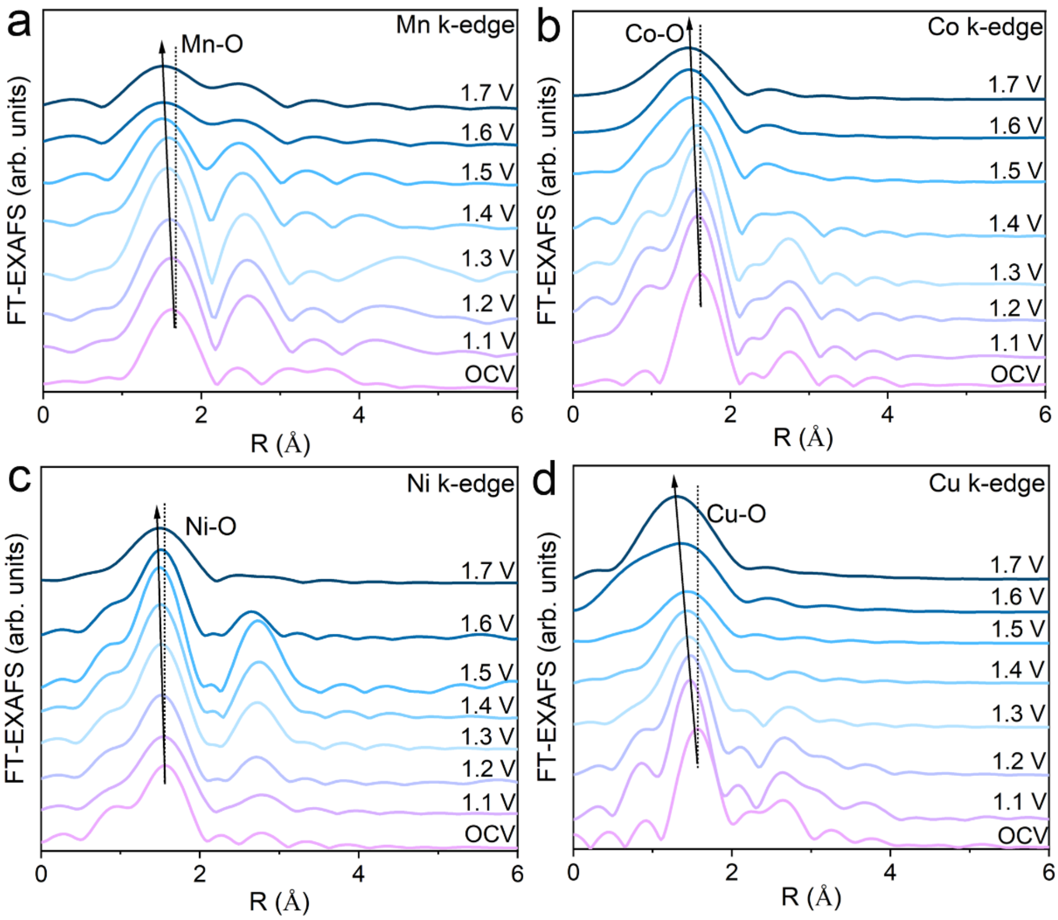

Fig. S42. Fourier transforms of operando Mn, Co, Ni and Cu K-edge EXAFS spectra of Na-HE LDH under applied potentials of 1.1–1.7 V vs. RHE in 1.0 M KOH.

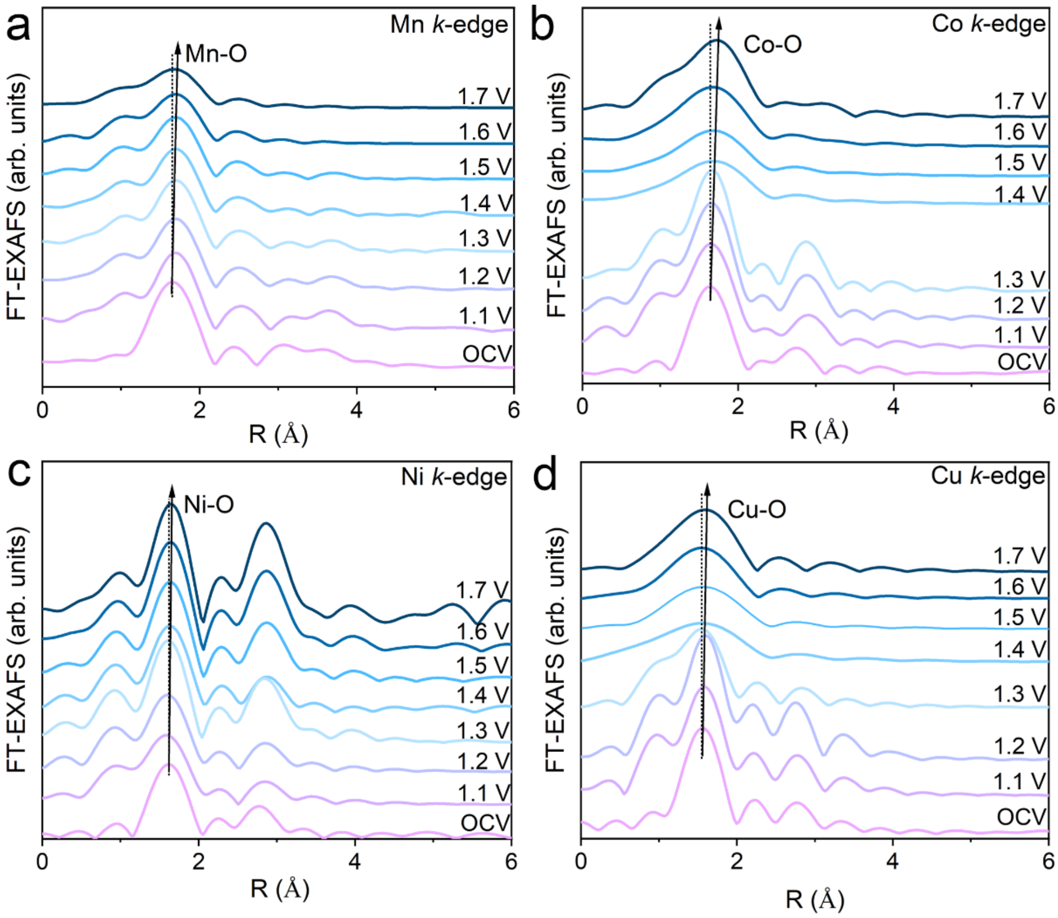

Fig. S43. Fourier transforms of operando Mn, Co, Ni and Cu K-edge EXAFS spectra of HE LDH under applied potentials of 1.1–1.7 V vs. RHE in 1.0 M KOH.

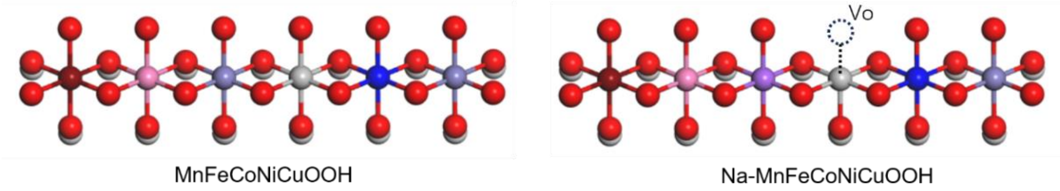

Fig. S44. The theoretical models of HEOOH and Na-HEOOH (crimson: Mn, pink: Co, cyan: Fe, gray: Ni, purple: Na, red: O).

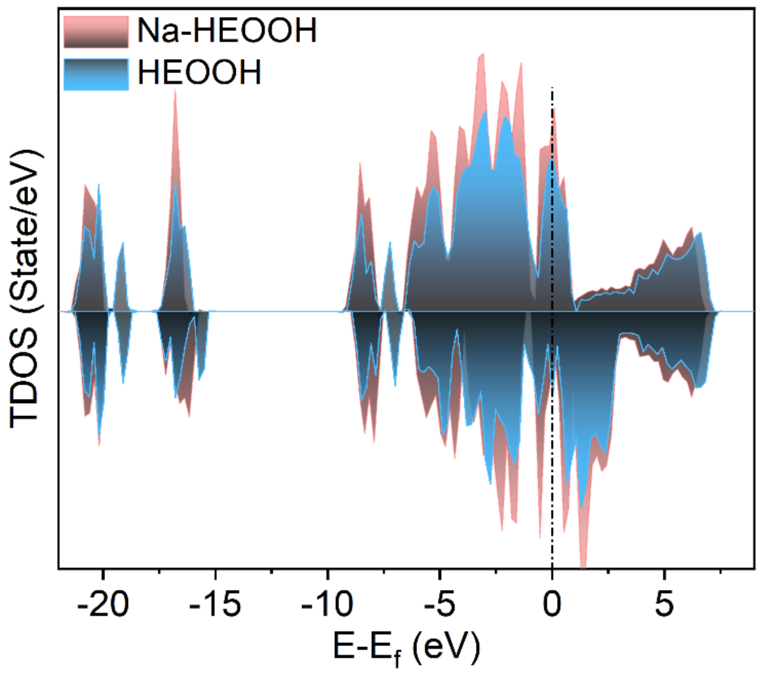

Fig. S45. Total density of states of Na-HEOOH and HEOOH.

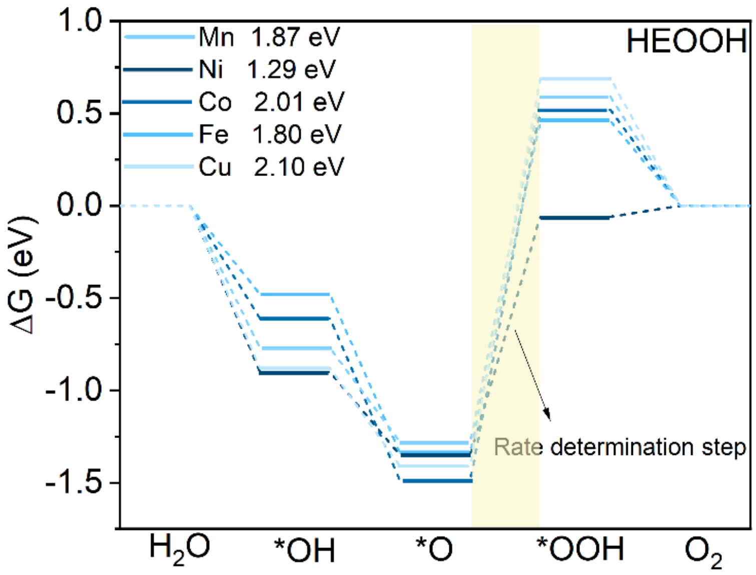

Fig. S46. Free energy profiles (AEM) for Mn, Ni, Co, Fe and Cu sites of HEOOH.

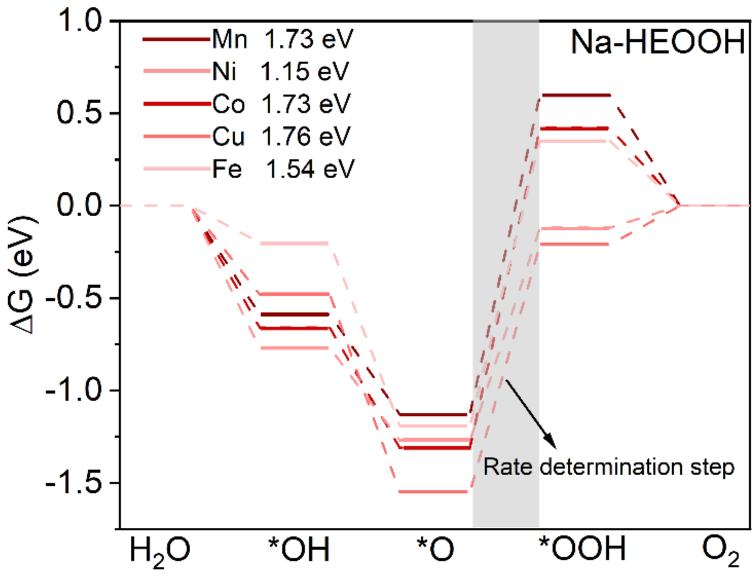

Fig. S47. Free energy profiles (AEM) for Mn, Ni, Co, Fe and Cu sites of Na-HEOOH.

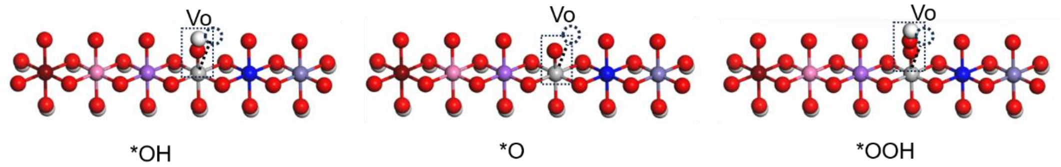

Fig. S48. The theoretical models of AEM on Na-HEOOH involved the adsorption of *OH, *O, *OOH (Ni is active site). (crimson: Mn, pink: Co, cyan: Fe, gray: Ni, purple: Na, red: O, white: H).

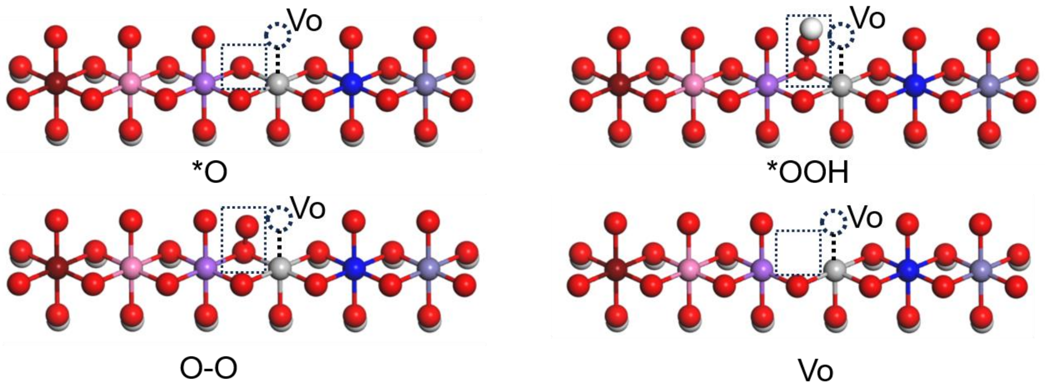

Fig. S49. The theoretical models of LOM on Na-HEOOH involved the adsorption of *O, *OOH, O-O, Vo. (crimson: Mn, pink: Co, cyan: Fe, gray: Ni, purple: Na, red: O, white: H).

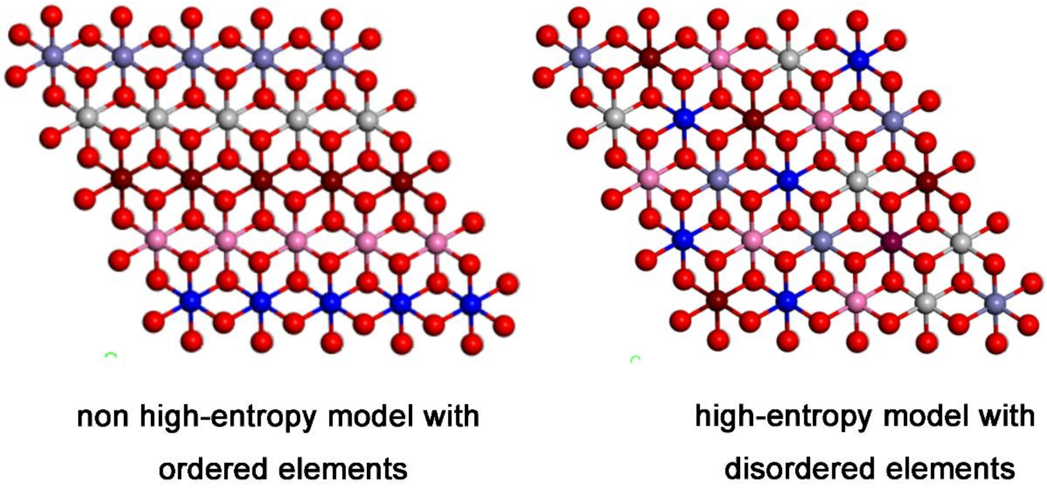

Fig. S50 non-high-entropy model with ordered 3d transition metals and high-entropy model with disordered 3d transition metals (crimson: Mn, pink: Co, cyan: Fe, gray: Ni, purple: Na, red: O, white: H).

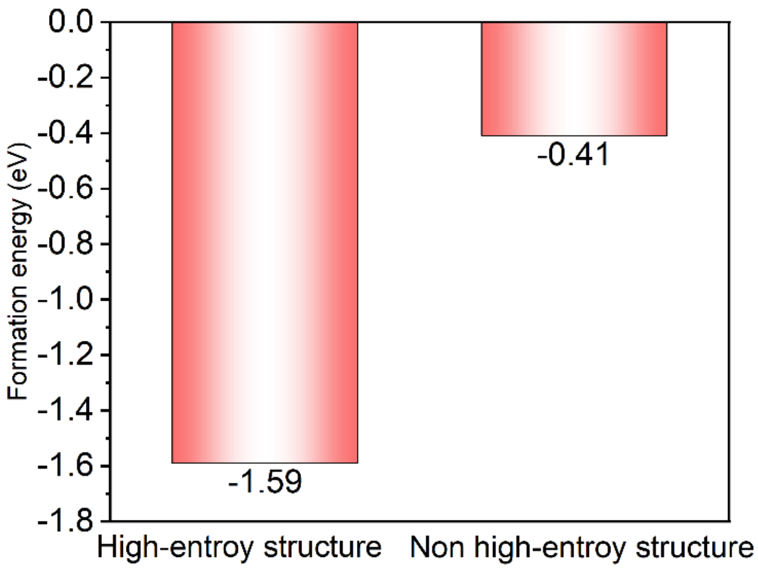

Fig. S51. The formation energy of high-entropy structure and non-high-entropy structure.

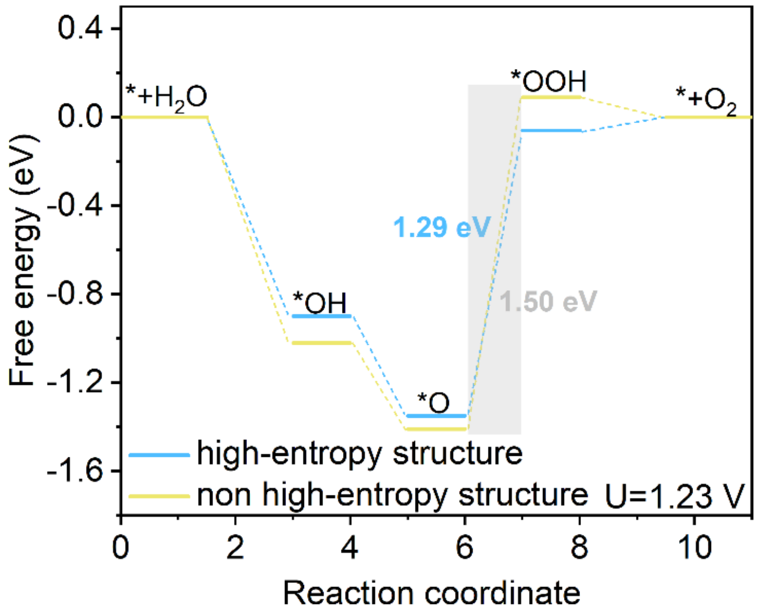

Fig. S52. Computed free energies (ΔG) of OER based on AEM of high-entropy structure and non-high-entropy structure.

Table S1. Compare activity, Tafel slope, ECSA and EIS for $Na_{0.025}$-HE LDH, $Na_{0.045}$-HE LDH and $Na_{0.065}$-HE LDH.

Catalyst	Activity (mV)	Tafel slope (mV dec^{-1})	ECSA (cm^2)	EIS (Ω)
$Na_{0.025}$-HE LDH	232	38.5	105	4.9
$Na_{0.045}$-HE LDH	176	29.6	62.5	2.8
$Na_{0.065}$-HE LDH	277	41.1	150	5.6

Table S2. EDS results (at%) for HE LDH and Na-HE LDH.

Sample	Mn	Fe	Co	Ni	Cu	Na	O
HE LDH	4.31	3.88	4.16	3.54	3.38	-	80.73
Na-HE LDH	4.23	3.67	4.24	3.62	3.48	0.19	80.57

Table S3. Fitting result of FT-EXAFS curves.

	Bond type	N^a	R^b (Å)	σ^{2c} ($Å^2$)	$\Delta E_0{}^d$ (eV)	R factor[e]
Na-HE LDH	Ni-O	5.08	2.04	0.0054	-2.06	0.0075
HE LDH	Ni-O	6.06	2.04	0.0094	-1.80	0.0113

[a] Coordination numbers. [b] Bond distance. [c] Debye-Waller factors. [d] Inner potential correction. [e] Goodness of fit. In term of NiO, the data range used for data fitting in k-space (Δk) and R-space (ΔR) are 2-10.3 $Å^{-1}$ and 1-2.1 Å, respectively. In terms of Na-HE LDH and HE LDH, the data range used for data fitting in k-space (Δk) and R-space (ΔR) are 2-9 $Å^{-1}$ and 1.1-2.5 Å, respectively.

Table S4. Comparison of OER performances of Na-HE LDH with previously reported well-performed OER electrocatalysts.

Catalyst	η@10mA cm^{-2} (mV)	η@50 mA cm^{-2} (mV)	η@100 mA cm^{-2} (mV)	Tafel (mV dec^{-1})	Loading (mg cm^{-2})	Electrolyte	Ref
Na-MnFeCoNiCu LDH (Na-HE LDH)	176	228	263	29.6	~1	1M KOH	This work
(Co, Cu, Fe, Mn, Ni)$_3$O$_4$/multi-walled carbon nanotubes	350	-		59.5	-	1M KOH	1
CV-activated MnFeCoNi HEA	302	-		83.7	2.6	1M KOH	2
FeCoNiCrNb$_{0.5}$	288	-		27.7	-	1M KOH	3
HF-treated CoFeNi	265	-		56.8	-	1M KOH	4
Fe-Cr-Co-Ni-Cu HE-LDHs-Ar-20	330	-		63.7	-	1M KOH	5
(CoNiMnZnFe)$_3$O$_{3.2}$	336	-		47.51	-	1M KOH	6
La$_5$(CrMnFeNi)$_2$Co	380	-		51.2	-	1M KOH	7
CoCrFeMnNi glycerate	229	-		40	2.5	1M KOH	8
NiCoFeCrMo-based HEH	292	-		54.31	-	1M KOH	9
FeCoNiMn	266	-		-	-	1M KOH	10
FeNiCoAl	225	-		68.14	0.35	1M KOH	11
CuCoNiFeMn high-entropy-alloy -60 h		479	-	-	-	1M KOH	12
V$_{1.0}$- CuCoNiFeMn High-Entropy Alloy		370	-	-	-	1M KOH	13
FeCoNiCuMn@CF HEAs			260	28	1	1M KOH	14

Au_{SA}-MnFeCoNiCu LDH	213	260	27.5	1	1M KOH	[15]

Table S5. The loading amounts of Na-HE LDH and HE LDH.

Sample	Loading amounts (mg cm^{-2})			
	1st	2nd	3nd	Mean
Na-HE LDH	0.98	0.94	1.12	1.01
HE LDH	0.93	1.05	1.02	1.00

Table S6. Stability comparison of Na-HE LDH and other LDHs/oxyhydroxides OER catalysts.

Sample	Current density (mA cm^{-2})	Time (h)	Decay	Ref.
**Na-HE LDH**	**500**	**1000**	**7%**	**This work**
Ir/CoFe-LDH/rGO	200	1000	-	[16]
CoCr LDH-2 light	15	15	-	[17]
NiFe LDH-PANI	20	15	little degradation	[18]
Fe-NiCo-LDH	500	500	negligible decay	[19]
NiFe-LDH@IF-200-72	100	192	-	[20]
S-NiFe LDH	100	190	-	[21]
$Co_{2.8}$,$W_{3.8}$-NiFe LDH	500	200	negligible decay	[22]
5% Ce-doped LDHs	10	24		[23]
NiFe-LDH/Ni4Mo	100	60	Nearly unchanged	[24]
1D-Fe_3O_4@C@NiFe-LDH-1	30	24	13.6%	[25]
$Ni_{0.75}V_{0.25}$ LDH	10	100	0.03%	[26]
NiFe/Co_3O_4@NF	210	24	4.4%	[27]
NiIr LDH	500	650	nearly unchanged	[28]
^{SA}Ru/NiFeLDH	10	7	2.7%	[29]

Table S7. Cost analysis of Na-HE LDH.

	Reagent	Unit Price (USD/kg)	Used Amount (mmol)	Used Amount (g)	Cost per Batch (USD)
Na-HE LDH	$Ni(NO_3)_2·6H_2O$	295	0.45	0.1309	0.0386
	$Fe(NO_3)_2·9H_2O$	191	0.45	0.1818	0.0347
	$Co(NO_3)_2·6H_2O$	564	0.45	0.1310	0.0739
	$Mn(NO_3)_2·4H_2O$	244	0.45	0.1130	0.0276
	$Cu(NO_3)_2·3H_2O$	408	0.45	0.1087	0.0443
	NH_4F	472	4	0.1482	0.0699
	urea	121	10	0.6006	0.0727
	NaCl	62	0.045	0.0026	0.0002
	Carbon cloth	14/(16*16 cm)	-	2*2 cm	0.2188
	Total	-	-	4 mg	**0.5807**
IrO_2	-	$702/g	-	10 mg	**7.02**

Note: All price information is from Sigma-Aldrich, as of April 19, 2025.

Table S8. Calculated ΔU values of HEOOH and Na-HEOOH.

	Mn	Fe	Co	Ni	Cu
HEOOH	3.837	3.830	3.812	3.873	3.827
Na-HEOOH	4.045	4.077	4.053	4.102	4.063

Table S9. Calculated LHB values (eV) of HEOOH and Na-HEOOH.

	Mn	Fe	Co	Ni	Cu
HEOOH	-2.854	-2.832	-2.842	-2.878	-2.860
Na-HE OOH	-2.989	-2.997	-2.969	-3.015	-2.977

Table S10. Calculated UHB values (eV) of HEOOH and Na-HEOOH.

	Mn	Fe	Co	Ni	Cu
HEOOH	0.983	0.998	0.970	0.995	0.967
Na-HE OOH	1.056	1.080	1.084	1.087	1.086

Table S11. Calculated Gibbs free energy (ΔG) values (U=1.23 V) in AEM on the Mn, Fe, Co, Ni, Cu sites of Na-HEOOH and HEOOH.

	site	ΔG_{*OH} (eV)	ΔG_{*O} (eV)	ΔG_{*OOH} (eV)
HEOOH	Mn	-0.77	-1.28	0.59
	Fe	-0.48	-1.34	0.46
	Co	-0.61	-1.49	0.52
	Ni	-0.90	-1.35	-0.06
	Cu	-0.88	-1.41	0.69
Na-HEOOH	Mn	-0.59	-1.13	0.60
	Fe	-0.20	-1.19	0.35
	Co	-0.66	-1.31	0.42
	Ni	-0.77	-1.27	-0.12
	Cu	-0.48	-1.55	-0.21

Table S12. Calculated Gibbs free energy (ΔG) values in LOM of HEOOH and Na-HEOOH.

	ΔG_{*O} (eV)	ΔG_{*OOH} (eV)	ΔG_{Vo+O2} (eV)
HEOOH	1.31	1.05	0.23
Na-HEOOH	0.52	1.01	0.11

Supplementary References

1. Wang, D., *et al.* Low-temperature synthesis of small-sized high-entropy oxides for water oxidation. *J. Mater. Chem. A* **7,** 24211-24216 (2019).
2. Dai, W., Lu, T., Pan, Y. Novel and promising electrocatalyst for oxygen evolution reaction based on MnFeCoNi high entropy alloy. *Journal of Power Sources* **430,** 104-111 (2019).
3. Ding, Z., *et al.* High Entropy Intermetallic–Oxide Core–Shell Nanostructure as Superb Oxygen Evolution Reaction Catalyst. *Adv. Sustain. Syst.* **4,** 1900105 (2020).
4. Ma, P., *et al.* Hydroxylated high-entropy alloy as highly efficient catalyst for electrochemical oxygen evolution reaction. *Science China Materials* **63,** 2613-2619 (2020).
5. Gu, K., *et al.* Ultrathin defective high-entropy layered double hydroxides for electrochemical water oxidation. **60,** 121-126 (2021).
6. Zhang, Y., Dai, W., Zhang, P., Lu, T., Pan, Y. In-situ electrochemical tuning of (CoNiMnZnFe)3O3.2 high-entropy oxide for efficient oxygen evolution reactions. *J. Alloys Compd.* **868,** 159064 (2021).
7. Nguyen, T. X., Liao, Y.-C., Lin, C.-C., Su, Y.-H., Ting, J.-M. Advanced High Entropy Perovskite Oxide Electrocatalyst for Oxygen Evolution Reaction. *Adv. Funct. Mater.* **31,** 2101632 (2021).
8. Nguyen, T. X., Su, Y.-H., Lin, C.-C., Ruan, J., Ting, J.-M. A New High Entropy Glycerate for High Performance Oxygen Evolution Reaction. *Adv. Sci.* **8,** 2002446 (2021).
9. Zhang, T., *et al.* Boosting the oxygen evolution electrocatalysis of high-entropy hydroxides by high-valence nickel species regulation. *ChemComm* **58,** 7682-7685 (2022).
10. Zheng, S., *et al.* A feasible method for the fabrication of VAlTiCrSi amorphous high entropy alloy film with outstanding anti-corrosion property. *Appl. Surf. Sci.* **483,** 870-874 (2019).
11. Han, M., *et al.* Promoted self-construction of β-NiOOH in amorphous high entropy electrocatalysts for the oxygen evolution reaction. *Appl. Catal. B* **301,** 120764 (2022).
12. Sivanantham, A., Lee, H., Hwang, S. W., Ahn, B., Cho, I. S. Preparation, electrical and electrochemical characterizations of CuCoNiFeMn high-entropy-alloy for overall water splitting at neutral-pH. *J. Mater. Chem. A* **9,** 16841-16851 (2021).
13. Sivanantham, A., *et al.* Complementary Functions of Vanadium in Boosting Electrocatalytic Activity of CuCoNiFeMn High-Entropy Alloy for Water Splitting. **33,** 2301153 (2023).
14. Yu, C., *et al.* Electrodeposition of FeCoNiCuMn high-entropy alloy nanoparticles as efficient bifunctional electrolytic water catalyst. *Surfaces and Interfaces* **46,** 104084 (2024).
15. Wang, F., *et al.* Activating lattice oxygen in high-entropy LDH for robust and durable water oxidation. *Nat. Commun.* **14,** 6019 (2023).
16. Cao, J., *et al.* Improved Electrocatalytic Activity and Stability by Single Iridium Atoms on Iron-based Layered Double Hydroxides for Oxygen Evolution. *Angewandte Chemie International Edition* **62,** e202310973 (2023).
17. Meng, H., *et al.* Solar-boosted electrocatalytic oxygen evolution via catalytic site remodelling of CoCr layered double hydroxide. *Appl. Catal. B* **284,** 119707 (2021).
18. Zhang, J., Zhang, H., Huang, Y. Electron-rich NiFe layered double hydroxides via interface engineering for boosting electrocatalytic oxygen evolution. *Appl. Catal. B* **297,** 120453 (2021).
19. Zhang, H., *et al.* The Guest Doping Effects of Fe on Bimetallic NiCo Layered Double Hydroxide for Enhanced Electrochemical Oxygen Evolution Reaction: Theoretical Screening and Experimental Verification. *Adv. Funct. Mater.* **33,** 2304403 (2023).
20. Zhao, W., *et al.* NiFe Layered Double Hydroxides Grown on a Corrosion-Cell Cathode for Oxygen Evolution Electrocatalysis. **12,** 2102372 (2022).

21. Lei, H., *et al.* Promoting Surface Reconstruction of NiFe Layered Double Hydroxide for Enhanced Oxygen Evolution. *Adv. Energy Mater.* **12,** 2202522 (2022).
22. Shi, Y., *et al.* Dual Cocatalytic Sites Synergize NiFe Layered Double Hydroxide to Boost Oxygen Evolution Reaction in Anion Exchange Membrane Water Electrolyzer. *Adv. Energy Mater.* **n/a,** 2402046 (2024).
23. Zubair, M., *et al.* Vacancy Promotion in Layered Double Hydroxide Electrocatalysts for Improved Oxygen Evolution Reaction Performance. *ACS Catal.* **13,** 4799-4810 (2023).
24. Wu, F., *et al.* Engineering Lattice Oxygen Regeneration of NiFe Layered Double Hydroxide Enhances Oxygen Evolution Catalysis Durability. *Angewandte Chemie International Edition* **n/a,** e202413250 (2024).
25. Cao, W., *et al.* Regulating the Spin Polarization of NiFe Layered Double Hydroxide for the Enhanced Oxygen Evolution Reaction. *ACS Catal.* **14,** 3640-3646 (2024).
26. Chavan, H. S., *et al.* Designing and Tuning the Electronic Structure of Nickel–Vanadium Layered Double Hydroxides for Highly Efficient Oxygen Evolution Electrocatalysis. *ACS Catal.* **12,** 3821-3831 (2022).
27. Lv, J., *et al.* Constructing a Hetero-interface Composed of Oxygen Vacancy-Enriched Co3O4 and Crystalline–Amorphous NiFe-LDH for Oxygen Evolution Reaction. *ACS Catal.* **11,** 14338-14351 (2021).
28. You, H., *et al.* Monolayer NiIr-Layered Double Hydroxide as a Long-Lived Efficient Oxygen Evolution Catalyst for Seawater Splitting. *J. Am. Chem. Soc.* **144,** 9254-9263 (2022).
29. Yang, Y., *et al.* Enhancing Water Oxidation of Ru Single Atoms via Oxygen-Coordination Bonding with NiFe Layered Double Hydroxide. *ACS Catal.* **13,** 2771-2779 (2023).
